# *SLC4A4*, *FRAS1*, and *SULT1A1* Genetic Variations Associated With Dabigatran Metabolism in a Healthy Chinese Population

**DOI:** 10.3389/fgene.2022.873031

**Published:** 2022-05-13

**Authors:** Qiufen Xie, Yuan Li, Zhiyan Liu, Guangyan Mu, Hanxu Zhang, Shuang Zhou, Zhe Wang, Zining Wang, Jie Jiang, Xin Li, Qian Xiang, Yimin Cui

**Affiliations:** ^1^ Department of Pharmacy, Peking University First Hospital, Beijing, China; ^2^ Department of Pharmacy, The Third Hospital of Changsha, Changsha, China; ^3^ School of Pharmaceutical Sciences, Peking University Health Science Center, Beijing, China; ^4^ Department of Cardiology, Peking University First Hospital, Beijing, China; ^5^ Institute of Clinical Pharmacology, Peking University, Beijing, China

**Keywords:** dabigatran, genome-wide association analysis, candidate gene, wholeexome sequencing, pharmacokinetics, pharmacodynamics

## Abstract

**Background:** The purpose of this study was to identify genetic variations associated with the metabolism of dabigatran in healthy Chinese subjects, with particular focus given to pharmacokinetics (PK) and pharmacodynamics (PD).

**Methods:** Healthy Chinese adults aged 18–65 years with unknown genotypes from a bioequivalence trial were included according to the protocol registered at ClinicalTrial.org (NCT03161496). All subjects received a single dose (150 mg) of dabigatran etexilate. PK (main outcomes: area under the concentration-time, AUC_0-t_, of total and free dabigatran) and PD (main outcomes: anti-FIIa activity, APTT, and PT) parameters were evaluated. Whole-exome sequencing and genome-wide association analyses were performed. Additionally, candidate gene association analyses related to dabigatran were conducted.

**Results:** A total of 118 healthy Chinese subjects were enrolled in this study. According to the *p*-value suggestive threshold (1.0 × 10^−4^), the following three SNPs were found to be associated with the AUC_0–t_ of total dabigatran: *SLC4A4* SNP rs138389345 (*p* = 5.99 × 10^−5^), *FRAS1* SNP rs6835769 (*p* = 6.88 × 10^−5^), and *SULT1A1* SNP rs9282862 (*p* = 7.44 × 10^−5^). Furthermore, these SNPs were also found to have significant influences on the AUC_0–t_ of free dabigatran, maximum plasma concentration, and anti-FIIa activity (*p* < 0.05). Moreover, we identified 30 new potential SNPs of 13 reported candidate genes (*ABCB1*, *ABCC2*, *ABCG2*, *CYP2B6*, *CYP1A2*, *CYP2C19*, *CYP3A5*, *CES1*, *SLCO1B1*, *SLC22A1*, *UGT1A1*, *UGT1A9*, and *UGT2B7*) that were associated with drug metabolism.

**Conclusion:** Genetic variations were indeed found to impact dabigatran metabolism in a population of healthy Chinese subjects. Further research is needed to explore the more detailed functions of these SNPs. Additionally, our results should be verified in studies that use larger sample sizes and investigate other ethnicities.

## 1 Introduction

Oral direct thrombin inhibitor (DTI), a new type of direct oral anticoagulants (DOACs), has been increasingly used in recent years for the prevention of stroke and systemic embolism in cases of non-valvular atrial fibrillation (NVAF) and are currently recommended as the first line of medication-based treatment for this condition, according to American CHEST Guidelines (Lip et al., 2018). Dabigatran, which is converted by liver esterase carboxylesterase 1 (CES1) from the oral prodrug dabigatran etexilate, is the sole reversible and active DTI (Stangier and Clemens, 2009). It was first approved by the United States (US) Food and Drug Administration (FDA) in 2010 for stroke prevention in patients with NVAF, and subsequently launched in China in 2013 (Li et al., 2020). With the advantages of rapid absorption, high bioavailability, fixed-dose regimens, requiring no coagulation monitoring, and having no food and few drug interactions, dabigatran is becoming more widely used in clinical application (Kanuri and Kreutz, 2019; Wieland and Shipkova, 2019).

Although the above characteristics of dabigatran are either equivalent or superior to Vitamin k Antagonists (Connolly et al., 2009), more attention should be given to its relative high risk for bleeding. In the US, dabigatran was the 10th most implicated drug for adverse drug events in emergency department visits among older adults in 2013–2014 (Shehab et al., 2016). However, the risk that dabigatran poses for bleeding may depend on inter-individual (as opposed to intra-individual) and ethnic variations. Extensive clinical experiences and studies have already demonstrated that inter-individual variations regarding pharmacokinetics (PK) and pharmacodynamics (PD) do exist. For example, Stangier et al. (2007) reported that the inter-individual coefficient of variation (CV) in PK parameters of dabigatran was approximately 30%, and that the CV in PD parameters was <10%. Besides, Chan et al. (2015) demonstrated that inter-patient variability in dabigatran level (geometric CV: 51–64%) was greater than intra-patient variability (geometric CV: 32–40%). Many factors, including renal function, age, weight, sex, and food consumption, may influence the PK parameters of dabigatran (Reilly et al., 2014). Yet these factors alone do not fully explain inter-individual variation, whether in the case of healthy volunteers or enrolled patients (Fawzy and Lip, 2019; Wieland and Shipkova, 2019).

The Framingham heart study (O'Donnell et al., 2001) would suggest that genetic factors play a major role in determining coagulation function. Dabigatran etexilate is a substrate of p-glycoprotein (P-gp), which is encoded by the *ABCB1* gene and generally not metabolized by cytochrome oxidase P450 (CYP450), except at supra-therapeutic concentrations (Raymond et al., 2021). Besides activation by the CES1 enzyme as previously mentioned, dabigatran is also metabolized to four active metabolites by UDP-glucuronyltransferase (UGT) isoforms 1A9, 2B7, and 2B15 (<10%) (Raymond et al., 2021). So far, there have indeed been several previous pharmacogenomics studies of dabigatran that explore the effects of genetic variation on its PK/PD parameters and clinical outcomes (Supplementary 1, Supplementary Table S1). However, these studies were mainly focused on genetic polymorphisms of *ABCB1* (rs1045642, rs1128503, rs2032582, and rs4148738) and *CES1* (rs2244613, rs8192935, and rs7164787). Only two studies (Lähteenmäki et al., 2021; Liu et al., 2021) have explored the influences of other genes, such as *CYP2D6*, *CYP3A5*, *SLC22A1*, and so on. Furthermore, the results of previous studies have not been totally consistent (Ašić et al., 2018; Cullell et al., 2018; Xie et al., 2018; Kanuri and Kreutz, 2019; Raymond et al., 2021; Shnayder et al., 2021). Most analytical methods involved a candidate gene approach, and only one study applied genome-wide association (GWA) analysis (Paré et al., 2013). As stated in the GWA study (GWAS), the frequency of the C allele in *CES1* rs2244613 varied from 15% in Europeans to 60% in Asians according to data from the 1000 Genomes Project (accessed July 2012), which has been verified in a recent Asian study (Ji et al., 2021). Finally, of all previous studies, only three have investigated Asian populations (Chinese and Japanese) (Tomita et al., 2016; Ji et al., 2021; Liu et al., 2021), where only one analyzed PD parameters (Ji et al., 2021) and none performed GWA.

With this information in mind, the current fixed doses of dabigatran may not be applicable to all real-world subjects. Additionally, data on genetic polymorphisms in Chinese populations is insufficient, and their potential roles in influencing PK/PD and clinical outcomes of dabigatran application remain unclear. Our study carried out whole-exome sequencing and genome-wide association analyses in healthy Chinese subjects, thus aiming to explore the potential metabolic pathways of dabigatran. Through studying the associations between various biomarkers and PK/PD outcomes in a separate cohort of healthy volunteers, we attempted to discover novel genetic marks and their respective values of influence on individual anticoagulation therapy with dabigatran.

## Materials and Methods

### Study Design

This genome-wide association study was based on a bioequivalence (BE) trial conducted in China, which was mainly performed to assess the BE of domestic generic dabigatran etexilate capsules, with reference to the original product (brand name: Pradaxa^®^), in healthy subjects under fasting and fed conditions (Li et al., 2020). Enrolled subjects with unknown genotypes were required to be 18–65 years old, have a BMI ranging from 19 to 26 kg/m^2^, and meet all other inclusion and exclusion criteria following screening visits. This BE trial followed an open-label, single-center, randomized, single-dose, four-period crossover design. Eligible healthy participants were randomly divided into either a test-reference-test-reference sequence group or a reference-test-reference-test sequence group. Subjects received a single dose of dabigatran etexilate capsule (150 mg) with 240 ml of warm water after either being fed a standard high-fat meal (Li et al., 2020) within 30 min or fasting overnight for at least 10 h. Then, they were discharged 48 h after drug administration. Based on the BE trial, we added genotype and PD parameter tests that were conducted during any one of the two reference periods. Additionally, PK parameters used for subsequent matching analysis were also obtained during this time.

This protocol was approved by an independent ethics committee and the Institutional Review Board of Peking University First Hospital (Protocol Code: 2016[1236]; date of approval: 11 October 2017) and the Third Hospital of Changsha (Protocol Code: 2018-KL-012; date of approval: 5 July 2018). The ClinicalTrial.org registration number was NCT03161496. This study was conducted in accordance with both the Good Clinical Practice guidelines and the Declaration of Helsinki. Prior to the beginning of the study, all involved subjects were briefed on the purpose, duration, and potential risks of the study and provided written informed consent.

### Sample Collection

Serial blood samples used for PK parameter analysis were collected from the BE trial. Venous blood samples (4 ml) were collected before dosing and then at 17–18 time points after drug administration (Li et al., 2020). We also collected 2 ml blood samples for genotyping before dosing, and 2.7 ml blood samples for PD parameter tests at 0 and 2 h after administration.

For PK analysis, blood samples were collected in EDTA-K_2_ tubes and centrifuged for 10 min at 4°C and 1700 g within 1 hour of sampling. Plasma samples were transferred to cryovials and stored at −80°C until PK analysis. For PD analysis, blood samples were collected in sodium citrate (3.2% v/v) tubes and centrifuged for 15 min at room temperature and 2500 g within 1 hour of sampling. Plasma samples were transferred to cryovials that were stored at −80°C, and were subjected to PD analysis within 6 months of sampling. For genetic polymorphism testing, blood samples were collected in EDTA-K_2_ tubes, transferred to cryovials, and stored at below −60°C until genotype testing.

### Pharmacokinetic Evaluations

Plasma concentrations of total and free (nonconjugated) dabigatran from BE trial center samples were determined by a home-validated liquid chromatography-tandem mass spectrometry (LC-MS/MS) method, which has been previously published (Li et al., 2020). PK parameters for total and free dabigatran were determined by standard noncompartmental methods using a WinNonlin 7.0 (Pharsight Corporation, California, United States). Maximum concentration (C_max_) and the time to C_max_ (T_max_) were recorded from the observed data. Values for the terminal half-life (t_1/2_) and area under the curve (AUC) for plasma concentration at time zero versus the last measurable time point (AUC_0-t_) were calculated. AUC_0-t_(total), C_max_(total), T_max_(total), and t_1/2_(total) reflected the PK parameters of total dabigatran, while AUC_0-t_(free), C_max_(free), T_max_(free), and t_1/2_(free) represented that of free dabigatran. No significant matrix effect was observed. All samples were analyzed within their established storage stability periods.

### Pharmacodynamic Evaluations

The prothrombin time (PT), activated partial thromboplastin time (APTT), and levels of anti-FIIa activity were assessed using the Sysmex^®^ CS-2100i fully automated multiparameter hemostasis analyzer (Sysmex, Kobe, Japan) at Peking University First Hospital. PT and APTT were measured by validated Coagulation Method Assay Kits (Thromborel-S^®^ and Actin^®^, Siemens Healthcare Diagnostics Products GmbH, Marburg, Germany). During the 2018 International Council for Standardization in Hematology (ICSH) (Gosselin et al., 2018), anti-FIIa activity was recommended as a marker for the qualitative assessment of dabigatran–furthermore, the correlation between anti-FIIA activity and drug concentration (*p* < 0.001) was demonstrated in our previous study of Chinese patients (Liu et al., 2020). Anti-FIIa activity was measured using a validated Chromogenic Method Anti-FIIa Kit (HEMOCLOT Thrombin Inhibitors^®^, HYPHEN BioMed, Neuville sur Oise, France). Specific calibrators and controls were used for dabigatran (BIOPHEN dabigatran^®^ Calibrator and Control, HYPHEN BioMed, Neuville sur Oise, France). The results of the anti-FIIa assay were reported in dabigatran units (with limits of detection for dabigatran of 0–550 ng/ml). The two QC sample concentrations used were 24 and 399 ng/ml. The inter- and intra-day precision (RSD%) were measured as < 6.76% and 7.53%, whereas inter- and intra-day accuracy were determined to be 97.71% and 86.69%. Relative changes in PT and APTT were also used for PD assessments, which involved taking the ratio after administration to before administration (i.e., ∆PT_2h_ = PT_2h_/PT_0h_).

### Whole-Exome Sequencing

For DNA preparation, the quality of isolated genomic DNA was assessed by using the following three methods in combination: 1) DNA degradation and contamination were monitored on 0.8% agarose gels; 2) DNA purity was checked using the NanoPhotometer^®^ Spectrophotometer (IMPLEN, CA, United States); 3) DNA concentration was measured using a Qubit^®^ DNA Assay Kit in a Qubit^®^ 3.0 Flurometer (Invitrogen, United States).

For library preparation and sequencing, the whole exome library was prepared using the Agilent SureSelect^XT^ Human All Exon V6 Kit (Agilent Technologies Inc., United States) according to the manufacturer’s standard protocol. Briefly, qualified genomic DNA was fragmented to an average size of 200bp. The ends of the DNA fragments were subjected to end repair processes, followed by A-tailing and adapter ligation. DNA fragments with adapters ligated on both ends were selectively enriched by polymerase chain reaction (PCR). After PCR, library hybridization and exome capture were performed using biotin labeled probes and magnetic bead selection. Captured libraries were enriched and tagged by PCR to prepare for sequencing. The final libraries were quantified using a KAPA Library Quantification Kit (KAPA Biosystems, South Africa) and an Agilent 2100 Bioanalyzer. Paired-end sequencing (2 × 150 bp) was performed on an Illumina NovaSeq 6000 sequencer (Illumina, United States).

Variant filtering and prediction were performed on 347,010 single-nucleotide polymorphisms (SNPs) detected in exons, with 200 bp extensions on either end. SNPs were retained for association analysis when the missing rate was <10%, minor allele frequency was >5%, and Hardy-Weinberg equilibrium *p*-value was >10^–6^. Based on the high-quality data obtained after quality control, PCA conducted in PLINK 1.09 was used for stratified population assessment to remove outlier samples. Finally, 75,348 SNPs were tested for association with PK and PD parameters of dabigatran.

### Statistical Analysis

Genome-wide analyses of PK and PD parameters pertaining to dabigatran were performed by linear regression, assuming an additive genetic model, in PLINK 1.09. To account for possible population stratification, all analyses were adjusted for the first four genetic principal components. Covariates of PK and PD indexes were also further adjusted for age, sex, food, and high-density lipoprotein (HDL) to correct for residual population stratification (the effect of sex and food on metabolism is shown in Supplementary 1, Supplementary Table S2). These variables were chosen because they were associated with PK and PD parameters of dabigatran in univariate analyses. Analyses of non-imputed data were performed using the Gen ABEL package in R, whereas imputed data were analyzed using the Prob ABEL package. According to our original plan, the genome-wide *p*-value significance threshold was set at the Bonferroni limit of 6.6 × 10^–7^ in the discovery phase–SNPs reaching this threshold were taken forward for replication. The significance level for successful replication was set at *p* = 0.05/75,348 of SNPs for which replication was attempted. Following the genome-wide analyses of the merged and imputed discovery and replication phase data, follow-up analyses were performed by adding each top hit SNP as an additional covariate in GWA. This procedure was performed in a sequential manner until no significant genome-wide signals remained. Regional plots were generated with LocusZoom (Pruim et al., 2010) and all other figures were created in R. Continuous variables are presented as mean ± standard deviation (SD), and two-sided *p*-values < 0.05 were considered statistically significant.

### Candidate Gene Association Analysis

To extensively investigate the influences of previously reported candidate genes on PK and PD parameters of dabigatran in our study population, we also identified related genes and made detailed analyses of these genes. According to previous pharmacogenomic studies (Supplementary 1, Supplementary Table S1) and reviews (Raymond et al., 2021; Shnayder et al., 2021), the following 22 genes were selected: *ABCB1*, *ABCC2*, *ABCG2*, *CES1*, *CES1P2*, *CYP1A2*, *CYP2A6*, *CYP2B6*, *CYP2C8*, *CYP2C9*, *CYP2C19*, *CYP2J2*, *CYP2D6*, *CYP3A4*, *CYP3A5*, *CYP4F2*, *SLCO1B1*, *SLC22A1*, *UGT1A1*, *UGT1A9*, *UGT2B7*, and *UGT2B15*. According to the number of independent variables and whether the data were normally distributed, we used the Student’s t-test or one-way analysis of variance (ANOVA) and the Mann-Whitney or nonparametric Kruskal–Wallis H test to analyze the data. Age, sex, food, and HDL were adjusted for by multivariate linear regression. Two-sided *p*-values < 0.05 were considered statistically significant.

## Results

### Basic Characteristics and PK/PD Parameters

A total of 118 healthy Chinese subjects were enrolled, of which mean age and BMI were 24 years (range: 18–41 years) and 22.2 kg/m^2^ (range: 19.1–25.9 kg/m^2^), respectively. Basic participant characteristics are presented in Table 1. Among the subjects, 59 volunteers completed the study under the fasting condition and the remaining 59 under the fed condition. After a single dose (150 mg) of dabigatran, the PK and PD outcomes for all subjects were as follows: for total dabigatran, AUC_0–t_: 1247.43 ± 442.08 ng h/mL, C_max_: 139.99 ± 55.61 ng/ml, T_max_: 3.61 ± 2.09 h, and t_1/2_: 8.78 ± 1.33 h; for free dabigatran, AUC_0–t_: 1110.42 ± 404.57 ng h/mL, C_max_: 125.22 ± 48.26 ng/ml, T_max_: 3.65 ± 2.10 h, and t_1/2_: 8.57 ± 1.57 h.

**TABLE 1:** Basic characteristics of Chinese healthy volunteers.

Variables	Total (*n* = 118)
Sex(M/F)	85/33
Age (y)	22 (20, 26)
BMI (kg/m^2^)	22.05 (20.80.23.33)
CREA umol/L	68.08 ± 12.03
ALT IU/L	16.00 (11.00, 21.25)
AST IU/L	20.00 (17.00, 22.25)
ALP IU/L	63.00 (51.00, 73.25)
HGB g/L	151.00 (136.00, 158.00)
RBC 10^12/L	5.10 ± 0.49
TG mmol/L	0.79 (0.59, 1.21)
TCHO mmol/L	4.27 (3.90, 4.97)
LDL mmol/L	2.56 ± 0.68
HDL mmol/L	1.42 (1.28, 1.67)

For the continuous variables, data in normal distribution were shown as “mean ± SD”, and in skewed distribution were shown as “median (25, 75 percentiles)”.

M, male; F, female; BMI, body mass index; CREA, creatinine; ALT, alanine aminotransferase; AST, aspartate aminotransferase; ALP, alkaline phosphatase; HGB, hemoglobin; RBC, red blood cell; TG, triglyceride; TCHO, total cholesterol; LDL, low density lipoprotein; HDL, high density lipoprotein.

### Effect of Suggestive Genetic Variations on the Metabolism of Dabigatran

**FIGURE 1:**
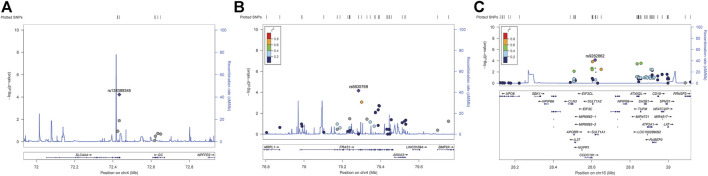
Regional association plots of suggestive SNPs on AUC_0–t_ of total dabigatran. **(A)** *SLC4A4* SNP rs138389345 **(B)** *FRAS1* SNP rs6835769 **(C)** *SULT1A* SNP rs9282862. Annotation: SNPs are shown according to their physical location and–log_10_ *p*-values for association. Also shown is the recombination rate in centimorgans per megabase (blue line) and the linkage disequilibrium (*r* ^2^) of each SNP with the SNP having the lowest *p*-value.

**FIGURE 2:**
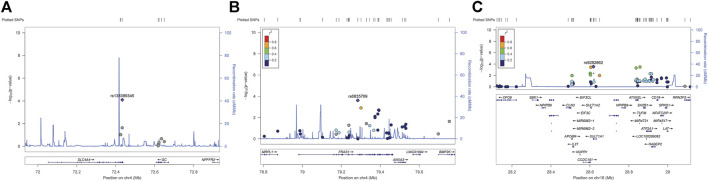
Regional association plots of suggestive SNPs on AUC_0–t_ of free dabigatran. **(A)** *SLC4A4* SNP rs138389345 **(B)** *FRAS1* SNP rs6835769 **(C)** *SULT1A* SNP rs9282862. Annotation: SNPs are shown according to their physical location and–log_10_ *p*-values for association. Also shown is the recombination rate in centimorgans per megabase (blue line) and the linkage disequilibrium (*r* ^2^) of each SNP with the SNP having the lowest *p*-value.

According to the genome-wide significance threshold (*p* = 6.6 × 10^–7^), no SNP was identified as significant. Considering the sample size of this exploratory experiment was small (*n* = 118), the *p*-value was adjusted to 1.0 × 10^−4^ for screening suggestive genetic variations on the main metabolic parameter of AUC_0–t_(total). Three SNPs, *SLC4A4* SNP rs138389345 (*p* = 5.99 × 10^−5^), *FRAS1* SNP rs6835769 (*p* = 6.88 × 10^−5^), and *SULT1A1* SNP rs9282862 (*p* = 7.44 × 10^−5^), exceeded our suggestive threshold for association with AUC_0–t_(total) (Manhattan plots and quantile-quantile plots presented in Supplementary 2, Supplementary Figures S1, S2). The related information of the three SNPs and their effects on outcomes are summarized in Table 2. Regional association plots within 500 kilobases of each SNP are shown in Figure 1. The minor alleles of *SLC4A4* SNP rs138389345 and *SULT1A1* SNP rs9282862 were both significantly associated with a lower AUC_0–t_(total), while the minor allele of *FRAS1* SNP rs6835769 was associated with a higher AUC_0–t_(total). The trends of influence on AUC_0–t_(free) for these suggestive SNPs could be also shown significantly (*p* < 1.0 × 10^−3^), with regional association plots presented in Figure 2. The effects of the three SNPs on C_max_(total), C_max_(free), and IIa2h were also similar (*p* < 0.05). However, these SNPs had no significant effect on the T_max_ or t_1/2_ of total or free dabigatran. As for the other PD parameters, the minor allele of *SLC4A4* SNP rs138389345 corresponded to a significantly lower APTT2h, and the minor allele of *SULT1A1* SNP rs9282862 had a significantly lower ∆PT2h (*p* < 0.05). However, *FRAS1* SNP rs6835769 was not associated with any index of APTT or PT.

**TABLE 2:** Effect of suggestive genetic variations on pharmacokinetic and pharmacodynamic parameters of dabigatran.

Gene	*SLC4A4*				*FRAS1*				*SULT1A1*		
SNP	rs138389345				rs6835769				rs9282862		
**CHR**	4				4				16		
**Func**	UTR3				Exonic				intronic		
**GENO**	23/64/31				4/41/73				0/56/62		
**Genotypes#**	**A1A1**	**A1A2**	**A2A2**	*p* **value**	**A1A1**	**A1A2**	**A2A2**	*p* **value**	**A1A2**	**A2A2**	*p* **value**
**AUC**_**0–t**_(**total) (ng·h*/*ml)**	1018.11 ± 269.56	1231.16 ± 429.25	1451.17 ± 471.41	**5.99E-05**	1947.95 ± 120.66	1337.98 ± 467.82	1158.19 ± 387.37	**6.88E-05**	1097.46 ± 337.20	1382.89 ± 476.91	**7.44E-05**
**AUC**_**0–t**_(**free) (ng·h*/*ml)**	926.80 ± 275.61	1084.37 ± 390.36	1300.43 ± 429.28	**8.09E-05**	1794.60 ± 55.65	1174.34 ± 418.92	1037.03 ± 360.93	**2.42E-04**	976.92 ± 329.75	1231.00 ± 424.29	**2.86E-04**
**C**_**max**_(**total) (ng*/*ml)**	111.28 ± 38.50	138.51 ± 52.52	164.35 ± 60.54	**1.70E-04**	225.18 ± 10.48	149.78 ± 57.19	129.83 ± 50.57	**2.52E-04**	126.61 ± 39.24	152.08 ± 64.32	**3.46E-03**
**C**_**max**_(**free) (ng*/*ml)**	103.56 ± 35.67	122.82 ± 43.75	146.22 ± 55.63	**2.40E-04**	199.53 ± 2.69	134.88 ± 50.78	115.71 ± 42.88	**1.23E-04**	114.56 ± 36.23	134.84 ± 54.90	**0.010**
**T**_**max**_(**total) (h)**	4.49 ± 2.75	3.42 ± 1.89	3.34 ± 1.68	0.557	2.25 ± 0.43	3.38 ± 1.19	3.81 ± 2.46	0.135	3.42 ± 1.97	3.78 ± 2.17	0.237
**T**_**max**_(**free) (h)**	4.50 ± 2.73	3.46 ± 1.87	3.41 ± 1.82	0.628	2.38 ± 0.65	3.40 ± 1.35	3.86 ± 2.42	0.124	3.41 ± 1.93	3.86 ± 2.21	0.162
**t**_**1*/*2**_ (total)*(h)	8.78 ± 1.85	8.88 ± 1.26	8.58 ± 0.94	0.166	8.41 ± 0.58	8.93 ± 1.43	8.72 ± 1.29	0.764	8.89 ± 1.60	8.68 ± 1.01	0.283
**t**_**1*/*2**_ (free)*(h)	8.47 ± 1.71	8.69 ± 1.69	8.39 ± 1.09	0.417	8.10 ± 0.53	8.67 ± 1.55	8.54 ± 1.60	0.945	8.64 ± 1.93*	8.50 ± 1.13	0.995
**IIa2h(ng/ml)**	56.19 ± 65.10	82.42 ± 71.88	121.58 ± 90.40	**2.93E-03**	226.01 ± 16.04	87.70 ± 84.83	79.96 ± 70.69	**6.97E-03**	76.69 ± 66.20	97.45 ± 88.32	**6.71E-03**
**APTT2h(s)**	34.19 ± 8.83	39.42 ± 9.77	42.16 ± 12.17	**0.034**	47.78 ± 3.49	38.80 ± 12.25	38.83 ± 9.68	0.761	38.74 ± 10.01	39.47 ± 11.16	0.233
**∆ APTT2h**	1.25 ± 0.36	1.38 ± 0.36	1.49 ± 0.43	0.200	1.84 ± 0.08	1.37 ± 0.42	1.36 ± 0.36	0.370	1.38 ± 0.37	1.39 ± 0.41	0.250
**PT2h(s)**	12.85 ± 2.79	12.61 ± 0.92	12.82 ± 1.22	0.538	14.00 ± 0.75	12.65 ± 1.05	12.68 ± 1.76	0.458	12.62 ± 1.92	12.80 ± 1.10	0.098
**∆ PT2h**	1.08 ± 0.26	1.09 ± 0.10	1.11 ± 0.14	0.878	1.22 ± 0.05	1.09 ± 0.11	1.09 ± 0.17	0.488	1.08 ± 0.18	1.11 ± 0.12	**0.044**

#*SLC4A4* SNP, rs138389345: A1= TG, A2= T; *FRAS1* SNP, rs6835769: A1= T, A2= C; *SULT1A1* SNP, rs9282862: A1= C, A2= T.

*1 subject in the group missed the value of t_1*/*2_(total) and t_1*/*2_(free).

SNP, single nucleotide polymorphism; CHR, chromosome; Func, the region of the genome where the mutation is; UTR, untranslated regions; A1, minor allele; A2, non-minor allele, GENO, Number of each genotype (A1A1/A1A2/A2A2); AUC, area under the plasma concentration-time curve; AUC_0-**t**_, area under the plasma concentration-time curve from 0 to the last measurable time point; C_max_, maximum plasma concentration; T_max_, time to peak concentration; t_½_, half-life; IIa2h: value of anti-IIa, at 2 h after dosing; APTT2h, value of activated partial thromboplastin time at 2 h after dosing; ∆ APTT2h, fold change in APTT, at post-dose 2 h to pre-dose 0h; PT2h, value of prothrombin time at 2 h after dosing; ∆ PT2h, fold change in PT, at post-dose 2 h to pre-dose 0 h. The bold values mean *p*-value < 0.05.

### Candidate Gene Association Analysis

For correlation analyses of candidate genes, the following 18 previously reported genes were finally included in this study: *ABCB1*, *ABCC2*, *ABCG2*, *CES1*, *CYP1A2*, *CYP2A6*, *CYP2B6*, *CYP2C8*, *CYP2C19*, *CYP2J2*, *CYP2D6*, *CYP3A5*, *CYP4F2*, *SLCO1B1*, *SLC22A1*, *UGT1A1*, *UGT1A9*, and *UGT2B7*. Among the 95 detected SNPs in this study, 10 SNPs of seven genes were reported in previous pharmacogenomic studies, including *ABCB1* SNP rs1045642, *ABCC2* SNPs rs2273697 & rs717620, *ABCG2* SNP rs2231142, *CES1* SNP rs2244613, *CYP2C19* SNP rs4244285, *CYP2D6* SNPs rs1065852 & rs28371725, and *SLCO1B1* SNPs rs2306283 & rs4149056. The effects of candidate genes on AUC_0–t_ and other PK/PD parameters are presented in Table 3 and Supplementary 1, Supplementary Tables S3, S4. Most of the results for these SNPs were consistent with previous studies, except those for three SNPs. *ABCC2* SNP rs2273697 and *SLCO1B1* SNP rs4149056 were significantly associated with AUC_0–t_ and C_max_, while *ABCB1* SNP rs1045642 had no influence on any of the PK or PD indexes in our study. Furthermore, 30 new potential SNPs of 13 genes were determined to be associated with PK/PD parameters. For the PK parameters, *ABCC2* SNP rs4148395, *SLCO1B1* SNPs rs2291075 & rs11045748, *CYP2B6* SNP rs56156262, *UGT1A1* SNP rs4148323, and *UGT1A9* SNP rs7586110 may have influences on AUC_0–t_ and C_max_; additionally, the last mentioned SNP may even be associated with T_max_. Furthermore, three SNPs (*ABCB1* rs2235015, *CYP2B6* rs434606, and *UGT1A1* rs4148327) were associated with C_max_, six (*ABCB1* rs2235047, *ABCG2* rs2231156, *UGT2B7* chr4:69879878 & rs115791839, and *UGT1A9* rs7563561 & rs7608175) with T_max_, and two (*CES1* rs56278207 and *CYP1A2* rs4646427) with t_1/2_. For the PD parameters, only two SNPs (*CYP2B6* rs2279342 and rs34433978) might influence IIa2h, where the latter has also been associated with APTT2h, PT2h, and ∆PT2h. *SLCO1B1* SNP rs11045748 might influence APTT2h, ∆APTT2h, and ∆PT2h. *CYP2C19* SNPs rs12769205, rs3758580, and rs4244285 might affect both APTT2h and PT2h. The remaining two (*ABCG2* rs2231138 and *SLC22A1* rs622591) and one (*CYP1A2* rs4646427) SNPs were associated with APTT2h and ∆APTT2h, respectively. At last, *CYP3A5* SNPs rs15524 and rs4646453 had effects on ∆PT2h.

**TABLE 3:** Positive effects of candidate genes on AUC_0–t_ of total and free dabigatran.

SNP	Gene	A1	A2	Number	GENO	AUC0–t(total) (ng·h/ml) (Mean ± SD)				AUC0–t(free) (ng·h/ml) (Mean ± SD)			
						A1A1	A1A2	A2A2	*p* value	A1A1	A1A2	A2A2	*p* value
**rs2273697#**	ABCC2	A	G	118	1/19/98	1841.88	1382.62 ± 565.47	1215.16 ± 403.91	0.016	1577.46	1249.36 ± 486.16	1078.72 ± 377.64	0.014
rs4148395	ABCC2	A	G	118	1/19/98	1841.88	1382.62 ± 565.47	1215.16 ± 403.91	0.016	1577.46	1249.36 ± 486.16	1078.72 ± 377.64	0.014
rs2291075	SLCO1B1	T	C	118	25/54/39	1115.84 ± 399.42	1226.24 ± 398.32	1361.13 ± 489.60	0.003	996.13 ± 359.02	1081.26 ± 362.54	1224.07 ± 451.62	0.005
**rs4149056#**	SLCO1B1	C	T	118	1/25/92	1286.96	1093.38 ± 355.33	1288.87 ± 453.93	0.019	1221.05	976.61 ± 338.97	1145.58 ± 413.15	0.042
rs11045748	SLCO1B7,SLCO1B1	T	G	118	3/20/95	1800.3 ± 193.01	1296.11 ± 455.59	1219.73 ± 430.00	0.020	1586.80 ± 187.34	1150.07 ± 434.64	1087.03 ± 390.71	0.024
rs34433978	CYP2B6,CYP2A13	C	CG	118	11/43/64	1223.24 ± 404.42	1332.82 ± 393.02	1194.22 ± 466.43	0.052	1083.22 ± 388.43	1209.71 ± 378.53	1048.39 ± 407.95	0.033
rs434606	CYP2B6,CYP2A13	A	G	118	15/60/43	1316.98 ± 448.90	1310.82 ± 449.57	1134.72 ± 399.31	0.050	1208.88 ± 427.43	1170.52 ± 398.33	992.22 ± 371.27	0.022
rs56156262	CYP2B6,CYP2A13	A	G	118	19/55/44	1335.32 ± 428.13	1277.95 ± 427.39	1171.34 ± 449.35	0.014	1203.27 ± 416.76	1146.34 ± 381.39	1025.42 ± 407.25	0.014
rs4148323	UGT1A1	A	G	118	0/35/83	—	1107.64 ± 327.95	1306.38 ± 467.36	0.030	—	978.76 ± 327.33	1165.94 ± 418.51	0.032
rs7586110	UGT1A8-10	G	T	118	4/42/72	921.44 ± 182.31	1105.51 ± 316.35	1348.33 ± 479.09	0.019	753.52 ± 171.09	998.81 ± 318.75	1195.36 ± 428.40	0.021

^#^The SNP, was detected in reported pharmacogenomic studies of dabigatran.

ABCC2, ATP Binding Cassette Subfamily C Member 2; SLCO1B1, Solute Carrier Organic Anion Transporter Family Member 1B1; SLCO1B7, Solute Carrier Organic Anion Transporter Family Member 1B7; CYP2B6, Cytochrome P450 Family 2 Subfamily B Member 6; CYP2A13, Cytochrome P450 Family 2 Subfamily A Member 13; UGT1A1, UDP, Glucuronosyltransferase Family 1 Member A1; UGT1A8, UDP, Glucuronosyltransferase Family 1 Member A8; UGT1A9, UDP, Glucuronosyltransferase Family 1 Member A9; UGT1A10, UDP, Glucuronosyltransferase Family 1 Member A10; AUC_0-t_, area under the plasma concentration-time curve from 0 to the last measurable time point. The bold values mean *p*-value < 0.05.

## Discussion

To the best of our knowledge, this was the first genome-wide association study to investigate genetic variations associated with pharmacokinetic and pharmacodynamic characteristics of dabigatran metabolism among healthy Chinese individuals. We performed whole-exome sequencing and candidate gene analysis to extensively explore and verify both novel and reported SNPs that may impact PK/PD parameters. First, we identified three suggestive SNPs (*SLC4A4* rs138389345, *FRAS1* rs6835769, and *SULT1A1* rs9282862) that were associated with the metabolism of dabigatran. AUC_0–t_(total), AUC_0–t_(free), C_max_(total), C_max_(free), and IIa2h values in the minor alleles of *SLC4A4* rs138389345 and *SULT1A1* rs9282862 were significantly lower compared to in their wild type counterparts. Conversely, these values were significantly higher in *FRAS1* SNP rs6835769. Additionally, *SLC4A4* rs138389345 and *SULT1A1* rs9282862 were found to have significant influences on APTT and PT, respectively. Moreover, through correlation analyses of reported candidate genes, we found inconsistencies in the results for three previously reported SNPs (*ABCC2* rs2273697, *SLCO1B1* rs4149056, and *ABCB1* rs1045642) regarding their associations with AUC_0–t_, C_max_, and PD indexes. Furthermore, we identified 30 new potential SNPs of 13 reported candidate genes in our study. *ABCB1*, *ABCC2*, *ABCG2*, *CYP2B6*, *CYP1A2*, *CES1*, *SLCO1B1*, *UGT1A1*, *UGT1A9*, and *UGT2B7* might influence PK parameters, while *ABCG2*, *CYP1A2*, *CYP2B6*, *CYP2C19*, *CYP3A5*, *SLCO1B1*, and *SLC22A1* might influence PD characteristics. Since our study was based on an original BE trial in healthy subjects, the accuracy of the PK/PD results was high and no known diseases or other factors affected drug metabolism. These same advantages of the previous study, which followed a similar experimental design (Liu et al., 2021), increased the quality and reliability of our results for determining the impact of various genetic polymorphisms on PK and PD parameters of dabigatran metabolism.

The major pharmacokinetic features of dabigatran etexilate include having a rapid absorption, low bioavailability (3–7%), variable peak concentration, steady state concentration attainment within two to 3 days, bi-exponential distribution phase, volume of distribution (50–70 L), 35% bind rate to plasma proteins, hepatic conjugation with glucuronide, and ability for renal elimination (80%) (Kanuri and Kreutz, 2019). As a BCS Class IIb drug, dabigatran etexilate exhibits high solubility at acidic pHs, but tends to precipitate after emptying from the stomach into the small intestine, leading to low bioavailability (Tsume et al., 2014). As previous reported, dabigatran is mainly eliminated in a non-metabolized form by urine (80%), and approximately 20% is conjugated to form dabigatran acylglucuronide eliminated in stools (Blech et al., 2008). While recent research by Park et al. (2021) with a new direct measurement method revealed that the portion of dabigatran conjugated to form dabigatran acylglucuronide would be much higher than previous results by the old indirect method. As renal excretion is the predominant elimination pathway for dabigatran, the medication is avoided in patients with impaired kidney function–especially those with end-stage renal disease (Ašić et al., 2018). Compared with the negligible role of phase I metabolic reactions, the formation of 1-O-acylglucuronide in phase II is the predominant route of dabigatran metabolism in humans (Blech et al., 2008). After conjugation with activated glucuronic acid, dabigatran acylglucuronide as the main metabolite possess the similar or even weaker anticoagulant effects to prolong APTT, PT, and TT compared with dabigatran (Blech et al., 2008; Kim et al., 2022).

Drug transporters, including members of the ATP-binding cassette (ABC) and solute carrier (SLC) superfamilies, are recognized as important determinants for governing the therapeutic response of many drugs (César-Razquin et al., 2015). *SLC4A4*, located in chromosome 4q13.3, encodes an anion exchange protein named electrogenic sodium bicarbonate cotransporter 1 (NBCe1) (Demirci et al., 2006). NBCe1 is involved in the regulation of bicarbonate secretion and absorption, as well as intracellular pH. Mutations in this gene are associated with proximal renal tubular acidosis, an extremely rare autosomal recessive syndrome involving ocular abnormalities and developmental delay (Dinour et al., 2004). *SLC4A4* SNP rs138389345 is a variant of *SLC4A4* in which a single G base is deleted (G > -). From the HPA RNA-seq normal tissue data, the *SLC4A4* gene is most expressed in the kidney. NBCe1 plays a major role in renal bicarbonate absorption via the proximal tubules and maintaining normal blood pH (Demirci et al., 2006). Abnormal function of NBCe1 can cause severe HCO3^-^ reabsorption and decrease blood HCO3^-^ concentration, resulting in acidic pH. Due to the strong pH-dependent solubility of dabigatran etexilate, with decreased solubility at basic pH values (Chai et al., 2016), it is reasonable to speculate the influence of *SLC4A4* genetic variance on dabigatran metabolism. In our study, *SLC4A4* SNP rs138389345 showed a significantly higher AUC_0-t_(total) (TGTG 1018.11 ± 269.56, TGT 1231.16 ± 429.25, TT 1451.17 ± 471.41, ngh/ml). Additionally, this SNP resulted in increased values for AUC_0-t_(free), C_max_(total), C_max_(free), IIa2h, and APTT2h.

The *FRAS1* gene, also known as KIAA1500, is located in chromosome 4q21.21, encodes the extracellular matrix (ECM) proteins, and regulates epidermal-basement membrane adhesion and organogenesis during development (Hoefele et al., 2013). *FRAS1* SNP rs6835769 involves the mutation of a C base to a T base (C > T) within the gene, which can lead to the A817V mutation at the protein level. *FRAS1* mutations have been confirmed to cause an extremely rare autosomal recessive genetic disorder named Fraser Syndrome (FRASRS), and seem to be responsible for non-syndromic unilateral renal agenesis (Hoefele et al., 2013). Furthermore, *FRAS1* mutations are associated with bone metabolism, where *FRAS1* may be involved in the metabolism of calcium and phosphorous (Reyer et al., 2019). While most studies of *FRAS1* mutation have focused on its role in the pathogenesis and prognosis of diseases (Zhan et al., 2014), there has only been one GWAS (Fridley et al., 2016) that investigated drug related variations. From a study of 74 invasive epithelial ovarian cancer patients observed at the Mayo Clinic, it was found that some genes including *FRAS1* may explain inter-patient variation in clinical response to platinum-taxane therapies. In our study, *FRAS1* SNP rs6835769 was associated with the PK parameters of AUC_0-t_ and C_max_, and followed the same trend of *SLC4A4* mutations. Perhaps the reasoning behind this is as follows: the *FRAS1* gene is secondarily expressed in the kidney, including in the glomerular podocytes and podocyte precursors (Pitera et al., 2008), while dabigatran is primarily excreted via the kidney. While this may provide preliminary insight into how *FRAS1* impacts dabigatran metabolism, the detailed mechanism must be further explored and investigated through *in vivo* and *in vitro* studies.

Drugs usually undergo two phases of metabolism in the body. The main purpose of phase II metabolic reactions is to combine reactions, increase drug polarity, and facilitate drug exclusion from the body. The most common phase II biotransformation enzymes include sulfotransferases (SULTs), N-acetyltransferases (NATs), and methyltransferases (MTs). SULTs utilizes 3′-phospho-5′-adenylyl sulfate (PAPS) as a sulfonate donor to catalyze the sulfate conjugation of a wide variety of acceptor molecules that bear a hydroxyl or an amine group (Daniels and Kadlubar, 2014). Sulfonation increases the water solubility of most compounds, and therefore their potential for renal excretion, but it can also result in bioactivation to form active metabolites. The *SULT1A1* gene locates in chromosome 16q11.2, and rs9282862 is a mutation of a T base to C base within a *SULT1A1* intron. Proteins encoded by *SULT1A1* are present in many human tissues, including the liver, kidney, and platelets (Raftogianis et al., 1997). The two most common *SULT1A1* variant alleles are 638G > A (formerly named rs9282861, now named rs1042028, SULT1A1*2) and 667A > G (rs1801030, SULT1A1*3). Previous reports have confirmed that these alleles have significantly different frequencies depending on race (Carlini et al., 2001). *SULT1A1* mutations could explain the wide range of individual differences in tamoxifen efficacy (Gjerde et al., 2008), and have also been shown to be involved in the heparan sulfate and heparin metabolism pathway (Jiang et al., 2011). Although no study (Shnayder et al., 2021) has investigated the impact of *SULT1A1* gene variants on the efficacy or toxicity of apixaban, another DOAC, *SULT1A1* is indeed involved in the conversion of approximately one fourth of the absorbed amount of Apixaban to inactive metabolites (Raymond et al., 2021). For the metabolism of Apixaban (leading to O-desmethyl-apixaban sulfate), SULT1A1 and SULT1A2 account for a quarter of its conversion to inactive metabolites (Kanuri and Kreutz, 2019). Several SNPs in linkage disequilibrium with each other and with SULT1A1*1 have also been identified in both the distal and proximal promoter region of *SULT1A1*, and are associated with platelet enzymatic activity (Ning et al., 2005; Lin et al., 2012). SNP rs9282862 is also in strong linkage disequilibrium with SULT1A1*1 (*r* ^2^ = 1.00, D´= 1.00), as seen in data from the Gambian Genome Variation Project (GGVP). *SLC4A4* SNP rs138389345 showed a significantly decreased AUC_0-t_(total) (TT 1382.89 ± 476.91, TC 1097.46 ± 337.20, ng h/ml), with similar trends observed in AUC_0-t_(free), C_max_(total), C_max_(free), IIa2h, PT2h, and ∆PT2h. Although this linkage disequilibrium needs to be verified in Asian populations, and UGTs were the main phase II enzymes involved, *SULT1A1* variation may have the potential to be a new biomarker for antithrombotic therapy–it would certainly be worthy to conduct more detailed studies to investigate this possibility.

For the reported candidate genes, 13 genes (*ABCB1*, *ABCC2*, *ABCG2*, *CYP2B6*, *CYP1A2*, *CYP2C19*, *CYP3A5*, *CES1*, *SLCO1B1*, *SLC22A1*, *UGT1A1*, *UGT1A9*, and *UGT2B7*) were found to have significant effects on drug metabolism. The most commonly reported SNPs, including *ABCB1* rs1045642 and *CES1* rs2244613, had no influence on any PK or PD parameters in our study, which was a finding consistent with Ji Q’s research (Ji et al., 2021) but not totally with Liu Y’s study of Chinese participants (Liu et al., 2021). As we know, UGTs are involved in the production of active metabolites in a small proportion of dabigatran (Ishiguro et al., 2014). Besides, polymorphisms of *CYP450* for isoenzymes *1A2*, *2C8*, *2C9*, *2C19*, and *2J2* have been shown to affect the pharmacokinetics of dabigatran, and especially the co-administration of DOACs with other drugs (Shnayder et al., 2021). Outside of the *ABCB1* and *CES1* genes, almost no study to date has focused on the associations between carriers of the *UGT* (including *UGT1A9*, *2B7*, and *2B15*), *CYP*, and *SLC* gene families and dabigatran metabolism in humans (Raymond et al., 2021; Shnayder et al., 2021). Zubiaur et al. (2020) investigated the influence of genetic polymorphisms in *UGT* and *CYP*, which encode for enzymes, and *SLC*, which encodes for transporters, on the pharmacokinetics and safety of dabigatran. In 107 healthy Caucasian and Latin-American volunteers, *UGT1A1* rs887829, *SLCO1B1*, and most *CYP* polymorphisms were found to have no influence in this regard. On the other hand, while *CYP2D6* poor metabolizers, *SLC22A1* haplotype, and *CYP3A5*-expressing subjects (*1/*1) had positive change. Lähteenmäki et al. (2021) investigated the effects of *ABCG2* and *CYP3A5* variants on bleeding and thromboembolic events in Finnish patients who were given dabigatran, where the results were all negative. In our study, the positive SNPs which had the most effects on PK or PD parameters included *SLCO1B1* rs11045748 (AUC_0-t_, C_max_, APTT2h, PT2h, and ∆PT2h), *SLCO1B1* rs2291075 & rs4149056 (AUC_0-t_ and C_max_), *UGT1A9* rs7586110 (AUC_0-t_, C_max_, and T_max_), *UGT1A1* rs4148323 & rs4148327, *ABCC2* rs2273697 & rs4148395 (AUC_0-t_ and C_max_), *CYP2B6* rs56156262 (AUC_0-t_ and C_max_), and *CYP2B6* rs34433978 (IIa2h, APTT2h, PT2h, and ∆PT2h). The reported positive SNPs of *CYP2D6*, *SLC22A1*, *CYP3A5*, and *ABCG2* were not consistent with previous studies (Zubiaur et al., 2020; Lähteenmäki et al., 2021). Accordingly, the impacts of all aforementioned SNPs should be verified in studies with larger sample sizes and that investigate other ethnicities.

There were a few key limitations to our study worth discussing. First, as the sample size was limited, no SNP was found to meet the genome-wide significance threshold. Therefore, future multi-center studies with larger sample sizes should be conducted. Second, not all potential SNPs could be detected by our genotyping method, such as the reported SNPs of *ABCB1*, *CES1*, *SULT1A1* rs9282861, and *UGT2B15* (Shnayder et al., 2021). Supplementary genome-wide detection or other forms of exploration should be performed to account for this. Third, as our study was only performed in healthy Chinese subjects, it should be replicated in another population–especially among patients and other ethnicities. As mentioned in the background, many factors may influence the metabolism of dabigatran and these indexes have different characteristics of distribution between healthy subjects and patients. Recent pharmacogenetic studies also showed different results between these two groups (Supplementary 1, Supplementary Table S1). Our findings Whether our findings can be replicated in patients or other ethnicities is worth exploring further. Finally, explorations into the functions of our newfound suggestive genes were mostly insufficient. More identification studies (both *in vivo* and *in vitro*) should be performed to document their detailed pathways in drug metabolism.

## Conclusion

Our study found that genetic variations do impact the metabolism of dabigatran in healthy Chinese populations. The suggestive genes of *SLC4A4*, *FRAS1*, and *SULT1A1* may be novel biomarkers and potential targets for anticoagulation therapy involving dabigatran. Furthermore, newly identified SNPs of 13 reported candidate genes (*ABCB1*, *ABCC2*, *ABCG2*, *CYP2B6*, *CYP1A2*, *CYP2C19*, *CYP3A5*, *CES1*, *SLCO1B1*, *SLC22A1*, *UGT1A1*, *UGT1A9*, and *UGT2B7*) may also affect dabigatran drug metabolism. Further studies are required to explore the more detailed functions of these SNPs. Additionally, the results of our study should be verified in a larger study among patients and to investigate their relationships with the clinical outcomes of antithrombotic therapy.

## Data Availability

The data presented in the study are deposited in the National Population Health Data Center (NPHDC) repository, accession number 10.12213/11.A0028.202009.338.V1.0 (https://www.ncmi.cn/phda/dataDetails.html?type=project_data&id=CSTR:A0006.11.A0028.202009.338.V1.0-V1.0). As the data of our paper was included in the whole data of the National Key R&D Program of China (No.2016YFC0904900), the name of deposited dataset was Chinese pharmacogenomics and precision medication gene sequencing dataset. The detailed information and the number of this program were also shown in NPHDC (link: https://www.ncmi.cn/phda/projectDataDetail.html?id=61d77ecc-5176-398e-a33a-c8458ad73b52).
