# Supplementary figures and images for: SLC4A4, FRAS1, and SULT1A1 Genetic Variations Associated With Dabigatran Metabolism in a Healthy Chinese Population
Source: Front Genet. 2022 May 13;13:873031. doi: 10.3389/fgene.2022.873031 (PMC9136018; doi:10.3389/fgene.2022.873031)

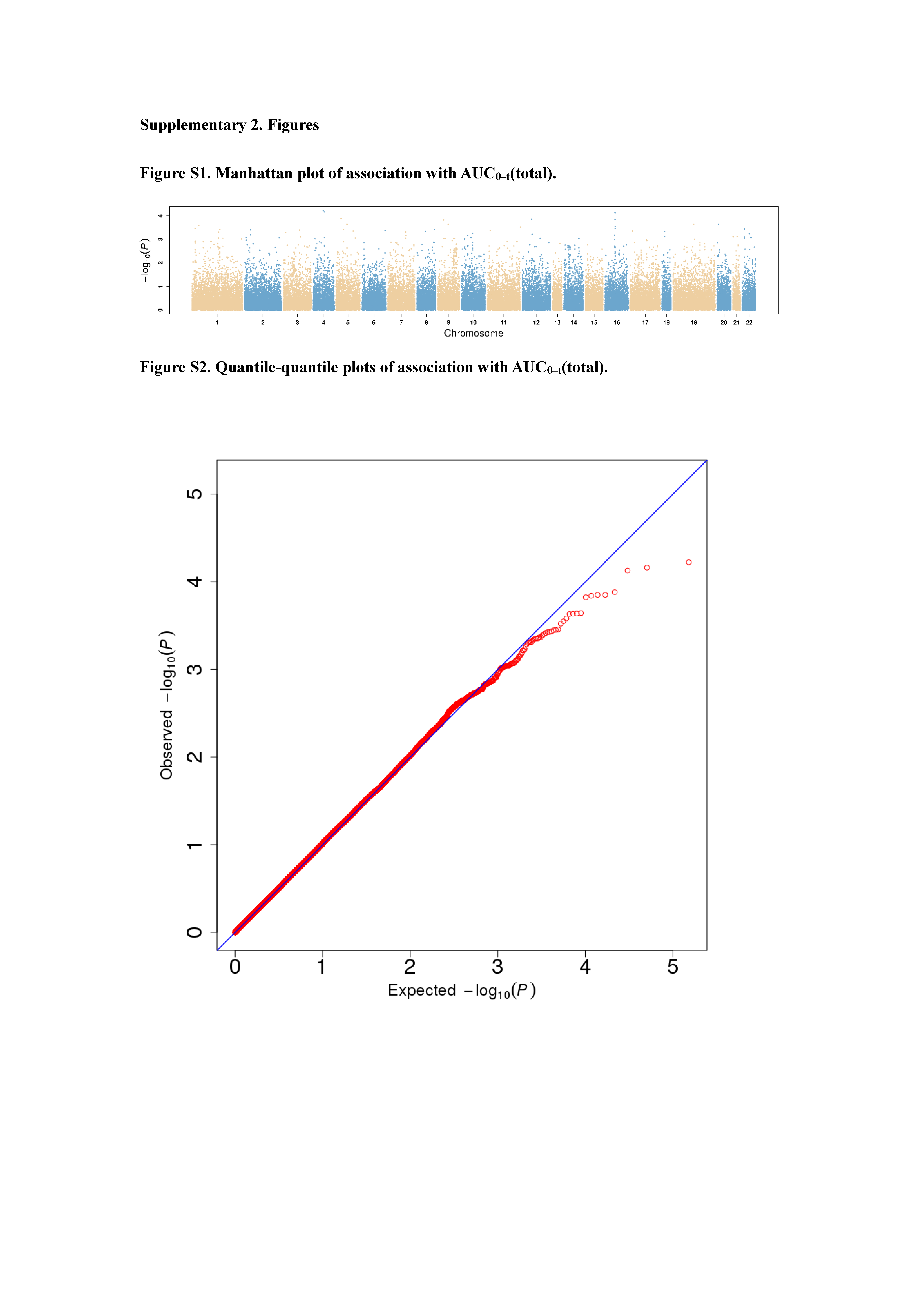

Supplement: Supplementary file 1 [file Image1.tif]
