# Supplementary material for: SLC4A4, FRAS1, and SULT1A1 Genetic Variations Associated With Dabigatran Metabolism in a Healthy Chinese Population
Source: Front Genet. 2022 May 13;13:873031. doi: 10.3389/fgene.2022.873031 (PMC9136018; doi:10.3389/fgene.2022.873031)
Supplement: Supplementary file 2 [file DataSheet1.docx]

**Supplementary 1. Tables**

**Table S1.** **Previous pharmacogenomic studies and candidate genes reported of dabigatran**

| **Literature** | **Sample size** | **Study population** | **Ethnicity** | **Genes** | **SNPs** | **PK/PD and Clinical Outcomes** | |
| --- | --- | --- | --- | --- | --- | --- | --- |
|  |  |  |  |  |  | **Indicators** | **Significant effects** |
| PMID: 23467860 | 1694 | NVAF Patients | European Caucasian | *ABCB1* | rs4148738, rs2235046, rs1128503, rs10276036, rs1202169, rs1202168, rs1202167, rs8192935 | C_trough_, C_peak_, bioavailability, volume of distribution, clearance, ischemic events, bleeding events | *CES1* rs2244613 was associated with C_trough_, and *ABCB1* rs4148738 and *CES1* rs8192935 were associated with C_trough_ at genome-wide significance (p < 9×10^−8^) with a gene-dose effect. |
|  |  |  |  | *CES1* | rs2244613, rs4122238, rs8192935 |  |  |
|  |  |  |  | *CES1P2* | rs4580160, rs4784563 |  |  |
| PMID: 24797400 | 52 | NVAF Patients | Caucasian | *ABCB1* | rs1045642, rs1128503, rs4148738, rs2032582 | PDC | No significant effect. |
|  |  |  |  | *CES1* | rs2244613, rs8192935, rs412223 |  |  |
| PMID: 27893182 | 60 | Healthy volunteer | Caucasian | *ABCB1* | rs1045642, rs2032582, rs1128503 | AUC_0-∞_, C_max_, T_max_, CV% | No significant effect. |
|  |  |  |  | *CES1* | rs2244613 |  |  |
| PMID:27526389 | 98 | NVAF Patients | Japanese | *ABCB1* | rs1045642, rs2032582, rs1128503 | C_trough_, 90min PDC, APTT, DD | No significant effect. Besides, this study only investigated the association between *ABCB1* and PDC, no with APTT and DD. |
| PMID:27261537 | 92 | AF Patients | Caucasian | *ABCB1* | rs4148738 | C_trough_, C_peak_ | The *CES1* rs8192935 significantly influenced the dabigatran trough concentrations (p = 0.023) and carriers of the T allele showed significantly lower concentrations than did carriers of the CC genotype. |
|  |  |  |  | *CES1* | rs2244613, rs8192935 |  |  |
| PMID:30100750 | 60 | Patients after total knee arthroplasty | Russian | *ABCB1* | rs1045642, rs4148738 | C_trough_, C_peak_, bleeding | *ABCB1* rs1045642 TT genotype was associated with higher C_peak_ and the higher risk of bleeding than the presence of CC genotype (p < 0.008). |
|  |  |  |  | *CES1* | rs2244613 |  |  |
| PMID:32564268 | 107 | Healthy volunteer | Caucasian  Latin-American | *ABCB1* | rs1045642, rs2032582, rs1128503, rs3842, rs10276036, rs7787082, rs4728709, rs10248420, rs10280101, rs12720067, rs11983225, rs4148737 | AUC/DW、C_max_ /DW、T_max_、t_½_,、Vd/F、Cl/F | 1.*CYP2D6* poor metabolizers were related to lower Cl/F (p = 0.049) and a tendency was observed towards higher AUC (p = 0.07), C_max_ (p = 0.062) and to lower Vd/F (p = 0.08).  2.*SLC22A1* haplotype was related to pharmacokinetic variability (p < 0.05).  3. *CYP3A5*-expressing subjects (*1/*1) were related to a higher t_1/2_ compared to *1/*3 and *3/*3 (ANOVA, p = 0.04, after Bonferroni post hoc, p = 0.09) |
|  |  |  |  | *ABCC2* | rs2273697, rs717620 |  |  |
|  |  |  |  | *CES1* | rs2244613, rs71647871, rs8192935 |  |  |
|  |  |  |  | *CYP1A2* | rs2069514, rs762551, rs2470890 |  |  |
|  |  |  |  | *CYP2A6* | rs28399433 |  |  |
|  |  |  |  | *CYP4F2* | rs2108622 |  |  |
|  |  |  |  | *CYP2B6* | rs3745274, rs3211371, rs32113719, rs2279345, rs2279343 |  |  |
|  |  |  |  | *CYP2C8* | rs11572103, rs10509681, rs1058930 |  |  |
|  |  |  |  | *CYP2C9* | rs1799853, rs1057910 |  |  |
|  |  |  |  | *CYP2C19* | rs4244285, rs4986893, rs28399504, rs12248560 |  |  |
|  |  |  |  | *CYP2D6* | rs35742686, rs3892097, rs5030655, rs5030867, rs5030865, rs5030656, rs1065852, rs5030865, rs28371706, rs28371725 |  |  |
|  |  |  |  | *CYP3A4* | rs35599367, rs55785340, rs4646438 |  |  |
|  |  |  |  | *CYP3A5* | rs776746, rs10264272 |  |  |
|  |  |  |  | *SLCO1B1* | rs4149056, rs2306283, rs4149015, rs11045879 |  |  |
|  |  |  |  | *SLC22A1* | rs72552763, rs12208357, rs34059508 |  |  |
|  |  |  |  | *UGT1A1* | rs887829 |  |  |
| PMID:32134727 | 96 | AF patients with CKD stage 3A-3B | Russian | *ABCB1* | rs1045642, rs4148738 | C/D ratio, bleeding | Patients with the rs2244613 *CC* genotype had lower C/D values (70% reduction in the mean C/D vs. *AA* genotype, p = 0.001). Low number of bleeding events precluded any analyses between genotypes and clinical outcomes. |
|  |  |  |  | *CES1* | rs2244613 |  |  |
| PMID:32961964 | 218 | AF Patients | Caucasian | *ABCB1* | rs1045642, rs4148738 | Non-major bleeding | No significant associations. A trend of association between TG haplotype with bleeding risk was observed. |
| PMID:33179295 | 198 | NVAF Patients | Chinese | *ABCB1* | rs1045642, rs4148738 | PDC, APTT, TT, bleeding | The minor allele (C) on the *CES1* rs8192935 was associated with PDCs and APTT values at trough level (P = .028 for peak PDC, P < 0.001 for trough PDC, P = .015 for APTT). The minor allele (A) on the *CES1* rs2244613 was associated with increased trough PDCs (ANOVA: P < .001; AA vs. CC, P < .001; CA vs. CC, P = 0.004) and higher risk for minor bleeding (p = 0.034). |
|  |  |  |  | *CES1* | rs2244613, rs8192935 |  |  |
| PMID:33935512 | 106 | Healthy volunteer | Chinese | *ABCB1* | rs1045642, rs4148738, rs2032582 | C_max_、T_max_、t_½_,、AUC_last_, AUC_0-∞_, DW ratio, AUC/DW, C_max_ /DW | Under fasting condition, there was no significant effect. Under fed condition, *CES1* SNP rs8192935 was associated with C_max_ and t_½_. *ABCB1* rs1045642 had a significant effect on T_max_. P values were all not shown in paper. |
|  |  |  |  | *CES1* | rs2244613, rs8192935 |  |  |
| PMID:34043814 | 340 | Patients* | Finnish | *ABCB1* | rs1045642, rs2032582, rs4148738, rs1128503 | Bleeding and thromboembolic events | No significant effect. |
|  |  |  |  | *ABCG2* | rs2231142 |  |  |
|  |  |  |  | *CES1* | rs2244613, rs8192935 |  |  |
|  |  |  |  | *CYP3A5* | rs776746 |  |  |

PK, pharmacokinetic; C_trough_, trough plasma concentration, 10–16 h after previous dose; C_peak_, peak plasma concentration, 1–3 h after the previous dose; C_max_, maximum plasma concentration; AUC_0–24h_, area under the curve from the time of dosing to 24 h after dosing; AUC_last_, area under the curve from the time of dosing to the last measurable concentration; AUC_0-∞_, area under the curve from the time of dosing to the last measurable concentration and extrapolated to infinity; T_max_, time to peak concentration; C/D ratio, trough plasma concentration/dose ratio; t_½_, half-life; CL/F, apparent oral clearance; Vd/F, apparent volume of distribution; AUC_0–12h_, area under the curve from the time of dosing to 12 h after dosing; AUC/DW, variables AUC_∞_ were divided by the dose/weight(DW) ratio; C_max_, variables C_max_ were divided by the DW; APTT, activated partial thromboplastin time; PDC, plasma dabigatran concentration; DD, D-dime; TT, thrombin time; AF, atrial fibrillation; NVAF, nonvalvular atrial fibrillation; CKD, chronic kidney disease

*Indication for dabigatran including: AF (78.8%); vascular disease (9.4%); pulmonary embolism (2.9%); stroke, cerebral infarction, atherosclerosis (4.7%); venous thrombosis (4.1%)

**Table S2. The effect of sex and food on the pharmacokinetic and pharmacodynamic parameters of dabigatran**

| **Groups** | | **Sex** | | | **Food** | | |
| --- | --- | --- | --- | --- | --- | --- | --- |
| **Parameters** | | **Male (n=85)** | **Female (n=33)** | **p value** | **Fasting (n=59)** | **Fed (n=59)** | **p value** |
| **PK** | **AUC_0–t_(total)(ng·h*/*ml)** | 1202.02±402.95 | 1364.42±518.42 | 0.073 | 1346.14±496.43 | 1148.73±357.75 | **0.015** |
|  | **AUC_0–t_(free)(ng·h*/*ml)** | 1063.24±381.31 | 1231.96±442.29 | **0.042** | 1157.38±459.81 | 1063.46±338.04 | 0.209 |
|  | **C_max_(total)(ng*/*ml)** | 135.79±50.72 | 150.81±66.21 | 0.184 | 155.94±59.70 | 124.04±46.43 | **0.004** |
|  | **C_max_(free)(ng*/*ml)** | 120.49±43.22 | 137.40±58.29 | 0.118 | 133.51±52.70 | 116.92±42.20 | 0.107 |
|  | **T_max_(total)(h)** | 3.41±1.84 | 4.14±2.59 | 0.181 | 2.23±0.61 | 4.99±2.13 | **< 0.001** |
|  | **T_max_(free)(h)** | 3.46±1.85 | 4.13±2.62 | 0.272 | 2.25±0.63 | 5.05±2.13 | **< 0.001** |
|  | **t_1_*_/_*_2_(total)*****(h)** | 8.90±1.22 | 8.46±1.58* | 0.067 | 9.15±1.38 | 8.41±1.18* | **0.002** |
|  | **t_1_*_/_*_2_(free)*****(h)** | 8.72±1.59 | 8.18±1.49* | 0.081 | 8.97±1.71 | 8.16±1.31* | **0.008** |
| **PD** | **IIa2h(ng/ml)** | 86.26±77.21 | 91.03±86.67 | 0.817 | 150.98±62.82 | 24.21±25.86 | **< 0.001** |
|  | **APTT2h(s)** | 38.69±10.15 | 40.22±12.05 | 0.651 | 47.12±7.89 | 31.13±6.16 | **< 0.001** |
|  | **∆ APTT2h** | 1.38±0.39 | 1.39±0.40 | 0.862 | 1.70±0.20 | 1.06±0.25 | **< 0.001** |
|  | **PT2h(s)** | 12.74±1.69 | 12.65±1.13 | 0.864 | 13.15±0.94 | 12.28±1.89 | **< 0.001** |
|  | **∆ PT2h** | 1.10±0.15 | 1.09±0.17 | 0.579 | 1.16±0.13 | 1.03±0.15 | **< 0.001** |

Data of the PK parameters were shown as “mean ± SD”

P value < 0.05 means the differences of the parameters among the groups were significant.

*1 subject in the group missed the value of t_1_*_/_*_2_(total) and t_1_*_/_*_2_(free).

AUC: area under the plasma concentration-time curve; AUC_0-_**_t_**: area under the plasma concentration-time curve from 0 to the last measurable time point; C_max_: maximum plasma concentration; T_max_: time to peak concentration; t_½_: half-life; IIa2h: value of anti-IIa at 2 h after dosing; APTT2h: value of activated partial thromboplastin time at 2 h after dosing; ∆ APTT2h: fold change in APTT at post-dose 2h to pre-dose 0h; PT2h: value of prothrombin time at 2 h after dosing; ∆ PT2h: fold change in PT at post-dose 2h to pre-dose 0h

**Table S3.** **Negative effects of candidate genes on AUC_0–t_ of total and free dabigatran**

| **SNP** | **Gene** | **A1** | **A2** | **Number** | **GENO** | **AUC_0–t_(total)(ng·h*/*ml) (Mean±SD)** | | | | **AUC_0–t_(free) (ng·h*/*ml) (Mean±SD)** | | | |
| --- | --- | --- | --- | --- | --- | --- | --- | --- | --- | --- | --- | --- | --- |
|  |  |  |  |  |  | **A1A1** | **A1A2** | **A2A2** | **p value** | **A1A1** | **A1A2** | **A2A2** | **p value** |
| **rs1045642#** | ABCB1 | A | G | 118 | 21/56/41 | 1295.81±486.70 | 1274.11±419.56 | 1186.22±435.93 | 0.340 | 1155.76±437.38 | 1132.14±375.03 | 1057.53±415.32 | 0.316 |
| rs2235013 | ABCB1 | T | C | 118 | 12/60/46 | 1281.86±402.33 | 1242.02±414.27 | 1245.51±480.27 | 0.494 | 1189.67±343.81 | 1109.21±371.45 | 1091.32±451.12 | 0.425 |
| rs2235015 | ABCB1 | A | C | 118 | 0/14/104 | / | 1367.40±329.94 | 1231.28±450.57 | 0.062 | / | 1229.78±316.70 | 1094.35±410.44 | 0.066 |
| rs2235033 | ABCB1 | G | A | 118 | 12/60/46 | 1281.86±402.33 | 1242.02±414.27 | 1245.51±480.27 | 0.494 | 1189.67±343.81 | 1109.21±371.45 | 1091.32±451.12 | 0.425 |
| rs2235047 | ABCB1 | C | A | 118 | 18/48/52 | 1223.84±321.09 | 1310.42±467.07 | 1197.46±443.07 | 0.569 | 1054.91±322.30 | 1151.61±421.92 | 1091.62±406.21 | 0.992 |
| rs2235048 | ABCB1 | G | A | 118 | 21/56/41 | 1295.81±486.70 | 1274.11±419.56 | 1186.22±435.93 | 0.340 | 1155.76±437.38 | 1132.14±375.03 | 1057.53±415.32 | 0.316 |
| rs4148734 | ABCB1 | A | G | 118 | 3/31/84 | 1308.33±171.11 | 1149.16±453.80 | 1281.53±436.25 | 0.132 | 1195.34±254.61 | 1056.70±414.79 | 1127.22±400.70 | 0.297 |
| rs3740066 | ABCC2 | T | C | 118 | 5/36/77 | 969.86±159.84 | 1245.20±427.26 | 1266.50±452.31 | 0.241 | 875.38±126.90 | 1125.36±391.61 | 1118.70±414.97 | 0.318 |
| rs3740073 | ABCC2 | T | C | 118 | 5/37/76 | 969.86±159.84 | 1230.98±427.67 | 1273.70±451.94 | 0.164 | 875.38±126.90 | 1115.10±390.02 | 1123.61±415.98 | 0.252 |
| **rs717620#** | ABCC2 | T | C | 118 | 5/32/81 | 969.86±159.84 | 1285.70±420.18 | 1249.45±453.47 | 0.408 | 875.38±126.90 | 1166.48±382.10 | 1102.78±415.98 | 0.555 |
| rs2231138 | ABCG2 | C | T | 118 | 1/18/99 | 2099.56 | 1386.29±541.05 | 1213.58±407.13 | 0.113 | 1906.98 | 1244.22±490.12 | 1078.05±372.89 | 0.069 |
| **rs2231142#** | ABCG2 | T | G | 118 | 6/52/60 | 1320.13±618.08 | 1239.75±437.79 | 1246.82±419.77 | 0.824 | 1169.92±579.10 | 1083.94±377.58 | 1127.42±401.03 | 0.661 |
| rs2231148 | ABCG2 | A | T | 118 | 9/45/64 | 1191.24±261.87 | 1226.15±438.75 | 1270.30±459.35 | 0.812 | 1064.95±237.38 | 1086.41±403.80 | 1133.70±418.82 | 0.991 |
| rs2231156 | ABCG2 | A | C | 118 | 4/39/75 | 1168.05±647.89 | 1325.80±464.91 | 1210.92±406.49 | 0.920 | 1008.31±619.99 | 1162.5±401.53 | 1088.79±385.44 | 0.856 |
| rs2231157 | ABCG2 | G | A | 118 | 13/47/58 | 1316.70±415.24 | 1155.68±356.21 | 1306.26±491.34 | 0.451 | 1226.57±377.33 | 1020.43±318.42 | 1157.31±452.02 | 0.723 |
| rs2231165 | ABCG2 | A | G | 118 | 5/40/73 | 1671.56±364.45 | 1212.07±481.73 | 1237.76±404.82 | 0.225 | 1545.74±300.83 | 1069.69±447.10 | 1102.92±364.22 | 0.273 |
| rs4148152 | ABCG2 | C | T | 118 | 13/59/46 | 1348.47±489.91 | 1260.03±453.87 | 1202.73±399.95 | 0.212 | 1195.12±481.51 | 1122.48±396.90 | 1071.02±380.75 | 0.287 |
| rs112236246 | CES1 | AC | A | 118 | 3/38/77 | 1105.40±152.83 | 1274.67±463.29 | 1239.53±434.64 | 0.534 | 1050.05±192.95 | 1131.67±456.73 | 1102.29±379.31 | 0.482 |
| **rs2244613#** | CES1 | T | G | 118 | 12/64/42 | 1193.14±383.14 | 1327.24±465.75 | 1141.34±387.47 | 0.161 | 1100.62±375.24 | 1173.49±427.10 | 1017.12±350.68 | 0.108 |
| rs2244614 | CES1 | A | G | 118 | 3/37/78 | 1105.40±152.83 | 1276.39±469.39 | 1239.16±431.86 | 0.472 | 1050.05±192.95 | 1132.94±462.80 | 1102.06±376.88 | 0.433 |
| rs2302719 | CES1 | G | T | 118 | 13/62/43 | 1172.50±374.99 | 1342.13±466.68 | 1133.55±384.24 | 0.112 | 1085.28±364.42 | 1188.22±428.82 | 1005.85±346.72 | 0.067 |
| rs3217164 | CES1 | T | TG | 118 | 3/36/79 | 1105.40±152.83 | 1274.67±475.75 | 1240.41±429.26 | 0.451 | 1050.05±192.95 | 1126.63±467.60 | 1105.33±375.59 | 0.451 |
| rs3815583 | CES1 | C | A | 118 | 17/67/34 | 1199.81±375.87 | 1227.48±446.87 | 1310.57±446.22 | 0.140 | 1045.17±331.11 | 1096.17±416.61 | 1171.12±400.25 | 0.125 |
| rs56278207 | CES1 | TA | T | 118 | 19/59/40 | 1276.01±396.67 | 1288.73±459.26 | 1172.95±421.13 | 0.320 | 1102.75±417.00 | 1143.56±405.47 | 1065.19±387.25 | 0.615 |
| rs12769205 | CYP2C19 | G | A | 118 | 16/42/60 | 1201.68±428.20 | 1128.18±422.91 | 1343.11±432.83 | 0.062 | 1080.00±377.17 | 999.85±415.01 | 1195.93±380.21 | 0.094 |
| rs17885098 | CYP2C19 | C | T | 118 | 2/17/99 | 1109.86±76.43 | 1240.57±372.29 | 1251.39±454.58 | 0.802 | 929.29±57.49 | 1107.22±349.87 | 1114.63±414.33 | 0.696 |
| rs3758580 | CYP2C19 | T | C | 118 | 16/42/60 | 1201.68±428.20 | 1128.18±422.91 | 1343.11±432.83 | 0.062 | 1080.00±377.17 | 999.85±415.01 | 1195.93±380.21 | 0.094 |
| **rs4244285#** | CYP2C19 | A | G | 118 | 15/43/60 | 1212.37±440.16 | 1126.16±418.17 | 1343.11±432.83 | 0.055 | 1089.68±387.60 | 998.34±410.27 | 1195.93±380.21 | 0.088 |
| **rs1065852#** | CYP2D6 | G | A | 117 | 24/55/38 | 1321.31±366.69 | 1220.75±421.85 | 1248.03±501.93 | 0.650 | 1164.57±343.85 | 1101.62±394.97 | 1095.05±447.08 | 0.705 |
| rs1080995 | CYP2D6 | G | C | 118 | 6/26/86 | 992.78±267.17 | 1197.47±406.71 | 1280.31±452.26 | 0.228 | 871.42±336.09 | 1062.95±335.59 | 1141.45±418.47 | 0.185 |
| rs1081003 | CYP2D6 | G | A | 118 | 25/55/38 | 1327.35±360.49 | 1210.51±422.45 | 1248.29±501.76 | 0.602 | 1174.09±340.11 | 1087.29±398.08 | 1102.01±441.56 | 0.671 |
| rs1135840 | CYP2D6 | C | G | 117 | 10/51/56 | 1242.20±340.12 | 1328.71±430.12 | 1180.21±454.66 | 0.343 | 1150.39±364.16 | 1180.99±386.57 | 1043.15±414.59 | 0.264 |
| rs16947 | CYP2D6 | A | G | 118 | 6/24/88 | 992.78±267.17 | 1213.48±419.11 | 1274.06±449.03 | 0.220 | 871.42±336.09 | 1066.28±348.03 | 1138.76±414.31 | 0.158 |
| **rs28371725#** | CYP2D6 | T | C | 117 | 1/15/101 | 933.09 | 1165.90±346.03 | 1265.90±453.03 | 0.726 | 800.62 | 1038.08±313.91 | 1126.53±415.28 | 0.636 |
| rs2291076 | SLCO1B1 | T | C | 118 | 9/43/66 | 1124.44±194.75 | 1294.10±479.12 | 1233.80±433.65 | 0.988 | 1038.26±280.86 | 1161.48±434.87 | 1087.00±391.45 | 0.715 |
| **rs2306283#** | SLCO1B1 | A | G | 118 | 10/41/67 | 1105.68±193.14 | 1299.51±483.11 | 1236.72±433.73 | 0.759 | 1037.63±266.46 | 1154.57±441.30 | 1094.27±392.19 | 0.996 |
| rs4149032 | SLCO1B1 | C | T | 118 | 23/54/41 | 1229.48±304.44 | 1191.27±464.26 | 1331.47±458.73 | 0.109 | 1114.25±328.31 | 1069.07±414.57 | 1162.74±418.63 | 0.342 |
| rs4149033 | SLCO1B1 | A | G | 118 | 0/28/90 | / | 1138.62±315.62 | 1281.29±467.17 | 0.056 | / | 1036.91±312.91 | 1133.29±424.40 | 0.156 |
| rs4149034 | SLCO1B1 | G | A | 118 | 24/53/41 | 1227.98±298.12 | 1191.23±468.62 | 1331.47±458.73 | 0.086 | 1106.95±323.30 | 1071.52±418.07 | 1162.74±418.63 | 0.281 |
| rs4149057 | SLCO1B1 | C | T | 118 | 9/42/67 | 1124.44±194.75 | 1287.07±482.60 | 1239.11±432.56 | 0.918 | 1038.26±280.86 | 1154.87±437.87 | 1092.25±390.86 | 0.826 |
| chr4:69879878 | UGT2A3,UGT2B7 | GA | G | 118 | 0/13/105 | / | 1259.22±469.43 | 1245.97±436.43 | 0.392 | / | 1177.81±435.91 | 1102.08±397.78 | 0.241 |
| rs115791839 | UGT2A3,UGT2B7 | A | T | 118 | 0/13/105 | / | 1259.22±469.43 | 1245.97±436.43 | 0.392 | / | 1177.81±435.91 | 1102.08±397.78 | 0.241 |
| rs12233719 | UGT2B7 | T | G | 118 | 1/41/76 | 1229.53 | 1327.04±484.01 | 1204.72±411.39 | 0.173 | 1088.20 | 1179.53±433.15 | 1073.43±383.16 | 0.221 |
| rs28365063 | UGT2B7 | G | A | 118 | 2/47/69 | 1703.76±474.23 | 1292.62±485.96 | 1203.42±393.25 | 0.155 | 1477.97±389.77 | 1153.60±434.26 | 1070.35±371.34 | 0.168 |
| rs4257713 | UGT2B7 | A | G | 118 | 4/51/63 | 1018.54±236.52 | 1192.68±394.90 | 1306.29±473.23 | 0.088 | 863.05±199.33 | 1066.96±355.46 | 1161.31±437.22 | 0.117 |
| rs5013211 | UGT2B7 | G | A | 118 | 11/56/51 | 1193.97±409.50 | 1202.57±401.71 | 1311.09±477.34 | 0.256 | 1043.11±389.17 | 1080.98±366.98 | 1157.26±436.83 | 0.354 |
| rs7438284 | UGT2B7 | A | T | 118 | 12/55/51 | 1202.57±393.91 | 1202.57±404.87 | 1311.09±477.34 | 0.272 | 1049.27±373.16 | 1080.33±370.27 | 1157.26±436.83 | 0.402 |
| rs7658752 | UGT2B7 | G | A | 118 | 12/55/51 | 1202.57±393.91 | 1202.57±404.87 | 1311.09±477.34 | 0.272 | 1049.27±373.16 | 1080.33±370.27 | 1157.26±436.83 | 0.402 |
| rs4646427 | CYP1A2 | C | T | 118 | 0/18/100 | / | 1299.04±369.21 | 1238.14±451.17 | 0.718 | / | 1124.13±324.21 | 1107.95±415.38 | 0.872 |
| rs1137115 | CYP2A6 | T | C | 116 | 15/32/69 | 1276.13±438.17 | 1222.07±367.30 | 1260.78±472.04 | 0.824 | 1107.20±400.30 | 1074.22±352.77 | 1135.94±425.66 | 0.995 |
| rs8192720 | CYP2A6 | A | G | 116 | 6/32/78 | 1097.36±412.22 | 1317.13±549.34 | 1237.30±386.34 | 0.780 | 952.13±377.16 | 1192.57±489.78 | 1096.00±359.17 | 0.919 |
| rs8192725 | CYP2A6 | A | G | 116 | 15/33/68 | 1276.13±438.17 | 1248.66±391.73 | 1248.44±464.33 | 0.703 | 1107.20±400.30 | 1099.46±375.57 | 1124.60±418.31 | 0.860 |
| rs8192726 | CYP2A6 | A | C | 116 | 2/22/92 | 1329.86±769.70 | 1279.84±340.77 | 1243.76±452.52 | 0.398 | 1225.89±681.09 | 1169.22±340.48 | 1099.87±408.92 | 0.279 |
| rs2279342 | CYP2B6 | T | A | 118 | 3/28/87 | 1413.02±502.68 | 1369.34±514.77 | 1202.49±401.35 | 0.082 | 1301.60±434.24 | 1219.99±478.23 | 1068.57±364.87 | 0.071 |
| rs8192719 | CYP2B6 | T | C | 118 | 3/46/69 | 1062.89±213.63 | 1289.83±407.78 | 1227.19±464.18 | 0.512 | 961.33±169.01 | 1159.56±377.12 | 1084.15±422.16 | 0.418 |
| rs35930845 | CYP2B6,CYP2A13 | G | C | 118 | 6/50/62 | 957.18±233.58 | 1236.06±401.64 | 1284.69±473.06 | 0.246 | 827.89±198.66 | 1077.20±363.95 | 1164.55±432.07 | 0.111 |
| rs3745275 | CYP2B6,CYP2A13 | A | G | 118 | 0/34/84 | / | 1307.57±487.51 | 1223.09±417.09 | 0.320 | / | 1188.28±435.63 | 1078.91±384.34 | 0.226 |
| rs3745276 | CYP2B6,CYP2A13 | A | G | 118 | 15/63/40 | 1145.99±480.73 | 1286.02±394.07 | 1224.70±483.41 | 0.736 | 1023.91±445.28 | 1133.02±357.05 | 1107.28±447.21 | 0.616 |
| rs3745277 | CYP2B6,CYP2A13 | A | G | 118 | 0/16/102 | / | 1264.64±513.18 | 1244.73±427.57 | 0.747 | / | 1119.66±453.15 | 1108.97±394.36 | 0.690 |
| rs7249735 | CYP2B6,CYP2A13 | C | A | 118 | 0/36/82 | / | 1271.32±497.29 | 1236.95±412.22 | 0.678 | / | 1157.64±442.34 | 1089.69±382.40 | 0.501 |
| rs1058932 | CYP2C8 | A | G | 118 | 19/51/48 | 1294.11±547.58 | 1176.09±451.84 | 1304.76±362.23 | 0.578 | 1137.95±469.44 | 1049.67±419.43 | 1164.08±343.09 | 0.490 |
| rs11572078 | CYP2C8 | TA | T | 118 | 19/50/49 | 1294.11±547.58 | 1180.52±455.24 | 1297.61±361.92 | 0.579 | 1137.95±469.44 | 1052.08±423.25 | 1159.28±341.20 | 0.488 |
| rs2071426 | CYP2C8 | C | T | 118 | 1/13/104 | 1033.43 | 1202.19±365.41 | 1255.15±449.91 | 0.318 | 871.80 | 1041.57±372.71 | 1121.32±406.83 | 0.296 |
| rs2275622 | CYP2C8 | C | T | 118 | 28/50/40 | 1194.08±486.81 | 1224.63±444.70 | 1313.28±389.34 | 0.177 | 1043.57±426.56 | 1092.53±411.84 | 1179.59±362.02 | 0.099 |
| rs2229189 | CYP2J2 | A | G | 118 | 0/13/105 | / | 1291.00±242.89 | 1242.04±458.48 | 0.295 | / | 1211.95±244.36 | 1097.85±416.60 | 0.160 |
| rs2271800 | CYP2J2 | C | A | 118 | 1/30/87 | 1193.49 | 1318.22±492.99 | 1223.65±420.35 | 0.695 | 938.96 | 1188.65±466.40 | 1085.42±376.87 | 0.537 |
| rs15524 | CYP3A5 | G | A | 118 | 14/55/49 | 1236.74±417.32 | 1205.19±448.08 | 1297.91±432.38 | 0.773 | 1122.9±377.32 | 1076.89±413.77 | 1144.49±394.35 | 0.823 |
| rs4646453 | CYP3A5,ZSCAN25 | A | C | 118 | 11/54/53 | 1223.59±445.54 | 1227.05±446.64 | 1273.15±431.02 | 0.723 | 1100.06±396.30 | 1100.99±412.47 | 1122.18±393.87 | 0.804 |
| rs2074900 | CYP4F2 | A | G | 118 | 12/46/60 | 1194.47±245.31 | 1223.08±447.13 | 1276.7±462.51 | 0.339 | 1104.56±265.05 | 1097.95±412.08 | 1121.15±417.96 | 0.642 |
| rs3093106 | CYP4F2 | C | T | 118 | 1/15/102 | 2033.91 | 1320.08±356.38 | 1229.04±445.33 | 0.151 | 1777.03 | 1170.3±323.86 | 1095.08±408.91 | 0.165 |
| rs3093160 | CYP4F2 | T | C | 118 | 1/15/102 | 2033.91 | 1320.08±356.38 | 1229.04±445.33 | 0.151 | 1777.03 | 1170.3±323.86 | 1095.08±408.91 | 0.165 |
| rs1867351 | SLC22A1 | C | T | 118 | 21/57/40 | 1361.94±436.74 | 1207.85±414.28 | 1243.72±466.70 | 0.482 | 1221.77±380.46 | 1072.59±399.78 | 1105.87±407.97 | 0.528 |
| rs2282143 | SLC22A1 | T | C | 118 | 0/23/95 | / | 1118.00±378.99 | 1278.77±448.20 | 0.386 | / | 974.27±345.13 | 1143.38±408.86 | 0.207 |
| rs35854239 | SLC22A1 | † | C | 118 | 7/51/60 | 1552.12±622.06 | 1197.31±439.04 | 1254.49±398.87 | 0.908 | 1361.34±511.73 | 1058.14±400.69 | 1125.59±377.16 | 0.859 |
| rs4646273 | SLC22A1 | A | G | 118 | 20/59/39 | 1377.22±442.02 | 1205.41±411.47 | 1244.45±467.62 | 0.407 | 1227.00±389.12 | 1075.48±399.20 | 1103.49±404.28 | 0.464 |
| rs622591 | SLC22A1 | C | T | 118 | 26/63/29 | 1241.97±402.16 | 1196.12±414.80 | 1363.81±500.41 | 0.573 | 1122.07±374.51 | 1065.06±395.15 | 1198.52±427.84 | 0.898 |
| rs628031 | SLC22A1 | A | G | 118 | 7/48/63 | 1552.12±622.06 | 1218.23±415.12 | 1235.83±421.38 | 0.767 | 1361.34±511.73 | 1073.26±375.40 | 1110.86±399.32 | 0.876 |
| rs683369 | SLC22A1 | G | C | 118 | 4/26/88 | 1423.04±250.83 | 1215.47±555.99 | 1248.90±404.90 | 0.922 | 1245.17±264.78 | 1076.24±490.10 | 1114.39±377.19 | 0.946 |
| rs2302538 | UGT1A1,3-10 | C | T | 118 | 1/13/104 | 1812.02 | 1340.02±483.02 | 1230.43±431.60 | 0.118 | 1238.31 | 1205.82±455.47 | 1097.27±395.90 | 0.321 |
| rs4148327 | UGT1A1,3-10 | C | T | 118 | 0/13/105 | / | 1448.13±555.26 | 1222.58±417.13 | 0.053 | / | 1256.74±485.65 | 1092.31±387.56 | 0.078 |
| rs12466997 | UGT1A3-10 | C | T | 118 | 5/38/75 | 1237.53±473.86 | 1278.15±530.97 | 1232.53±382.57 | 0.897 | 1132.85±428.67 | 1093.40±480.83 | 1117.55±354.57 | 0.613 |
| rs2361501 | UGT1A3-10 | T | A | 118 | 5/52/61 | 1202.25±447.53 | 1204.96±362.83 | 1287.35±492.86 | 0.998 | 1098.97±434.41 | 1067.62±344.84 | 1147.85±440.60 | 0.851 |
| rs869283 | UGT1A5-10 | A | G | 118 | 0/26/92 | / | 1117.31±234.57 | 1284.21±476.29 | 0.806 | / | 1022.61±243.35 | 1135.24±434.31 | 0.900 |
| rs7563561 | UGT1A7-10 | G | T | 118 | 6/47/65 | 958.28±166.99 | 1163.58±348.02 | 1334.76±491.13 | 0.296 | 855.95±201.93 | 1047.93±355.01 | 1179.10±431.52 | 0.294 |
| rs7608175 | UGT1A7-10 | G | C | 118 | 6/47/65 | 958.28±166.99 | 1163.58±348.02 | 1334.76±491.13 | 0.296 | 855.95±201.93 | 1047.93±355.01 | 1179.10±431.52 | 0.294 |

SNP: single nucleotide polymorphism; CHR: chromosome; BP: base pair; A1: minor allele; A2: non- minor allele; GENO: Number of each genotype (A1A1/A1A2/A2A2); ABCB1: ATP-binding cassette subfamily B member 1; CES1: Carboxylesterase 1; ABCC2: ATP Binding Cassette Subfamily C Member 2; ABCG2: ATP Binding Cassette Subfamily G Member 2; CYP2C19: Cytochrome P450 Family 2 Subfamily C Member 19; CYP2D6: Cytochrome P450 Family 2 Subfamily D Member 6; SLCO1B1: Solute Carrier Organic Anion Transporter Family Member 1B1; SLCO1B7: Solute Carrier Organic Anion Transporter Family Member 1B7; UGT2B7: UDP Glucuronosyltransferase Family 2 Member B7; UGT2A3: UDP Glucuronosyltransferase Family 2 Member A3; CYP1A2: Cytochrome P450 Family 1 Subfamily A Member 2; CYP2A6: Cytochrome P450 Family 2 Subfamily A Member 6; CYP2B6: Cytochrome P450 Family 2 Subfamily B Member 6; CYP2A13: Cytochrome P450 Family 2 Subfamily A Member 13; CYP2C8: Cytochrome P450 Family 2 Subfamily C Member 8; CYP2J2: Cytochrome P450 Family 2 Subfamily J Member 2; CYP3A5: Cytochrome P450 Family 3 Subfamily A Member 5; ZSCAN25: Zinc Finger And SCAN Domain Containing 25; CYP4F2: Cytochrome P450 Family 4 Subfamily F Member 2; SLC22A1: Solute Carrier Family 22 Member 1; UGT1A1: UDP Glucuronosyltransferase Family 1 Member A1; UGT1A3: UDP Glucuronosyltransferase Family 1 Member A3; UGT1A4: UDP Glucuronosyltransferase Family 1 Member A4; UGT1A5: UDP Glucuronosyltransferase Family 1 Member A5; UGT1A6: UDP Glucuronosyltransferase Family 1 Member A6; UGT1A7: UDP Glucuronosyltransferase Family 1 Member A7; UGT1A8: UDP Glucuronosyltransferase Family 1 Member A8; UGT1A9: UDP Glucuronosyltransferase Family 1 Member A9; UGT1A10: UDP Glucuronosyltransferase Family 1 Member A10; AUC0-t: area under the plasma concentration-time curve from 0 to the last measurable time point;

#The SNP was detected in reported pharmacogenomic studies of dabigatran

†: CTGGTAAGT

**Table S4.** **Effects of candidate genes on the other pharmacokinetic and pharmacodynamic parameters of dabigatran**

| **SNP** | **Gene** | **C_max_(total)** | **C_max_(free)** | **T_max_(total)** | **T_max_(free)** | **t_1_*_/_*_2_(total)** | **t_1_*_/_*_2_(free)** | **IIa2h** | **APTT2h** | **∆ APTT2h** | **PT2h** | **∆ PT2h** |
| --- | --- | --- | --- | --- | --- | --- | --- | --- | --- | --- | --- | --- |
|  |  | **p values** | | | | | | | | | | |
| **rs1045642#** | ABCB1 | 0.380 | 0.277 | 0.555 | 0.263 | 0.796 | 0.367 | 0.308 | 0.867 | 0.641 | 0.830 | 0.912 |
| rs2235013 | ABCB1 | 0.542 | 0.824 | 0.215 | 0.160 | 0.923 | 0.931 | 0.400 | 0.927 | 0.831 | 0.391 | 0.274 |
| rs2235015 | ABCB1 | **0.026** | **0.033** | 0.066 | 0.232 | 0.745 | 0.619 | 0.727 | 0.142 | 0.190 | 0.135 | 0.247 |
| rs2235033 | ABCB1 | 0.542 | 0.824 | 0.215 | 0.160 | 0.923 | 0.931 | 0.400 | 0.927 | 0.831 | 0.391 | 0.274 |
| rs2235047 | ABCB1 | 0.685 | 0.912 | **0.048** | **0.009** | 0.720 | 0.267 | 0.661 | 0.861 | 0.336 | 0.791 | 0.827 |
| rs2235048 | ABCB1 | 0.380 | 0.277 | 0.555 | 0.263 | 0.796 | 0.367 | 0.308 | 0.867 | 0.641 | 0.830 | 0.912 |
| rs4148734 | ABCB1 | 0.251 | 0.345 | 0.435 | 0.429 | 0.434 | 0.674 | 0.342 | 0.960 | 0.343 | 0.615 | 0.446 |
| **rs2273697#** | ABCC2 | **0.012** | **0.007** | 0.632 | 0.823 | 0.156 | 0.594 | 0.727 | 0.511 | 0.548 | 0.739 | 0.460 |
| rs3740066 | ABCC2 | 0.294 | 0.282 | 0.065 | 0.098 | 0.699 | 0.678 | 0.432 | 0.595 | 0.668 | 0.993 | 0.959 |
| rs3740073 | ABCC2 | 0.213 | 0.235 | 0.080 | 0.119 | 0.517 | 0.949 | 0.380 | 0.689 | 0.448 | 0.846 | 0.994 |
| rs4148395 | ABCC2 | **0.012** | **0.007** | 0.632 | 0.823 | 0.156 | 0.594 | 0.727 | 0.511 | 0.548 | 0.739 | 0.460 |
| **rs717620#** | ABCC2 | 0.519 | 0.514 | 0.184 | 0.262 | 0.701 | 0.574 | 0.385 | 0.453 | 0.451 | 0.940 | 0.904 |
| rs2231138 | ABCG2 | 0.207 | 0.063 | 0.587 | 0.853 | 0.832 | 0.985 | 0.106 | **0.039** | 0.703 | 0.231 | 0.300 |
| **rs2231142#** | ABCG2 | 0.726 | 0.536 | 0.073 | 0.069 | 0.825 | 0.788 | 0.974 | 0.554 | 0.733 | 0.137 | 0.511 |
| rs2231148 | ABCG2 | 0.423 | 0.497 | 0.070 | 0.077 | 0.381 | 0.415 | 0.793 | 0.966 | 0.069 | 0.896 | 0.784 |
| rs2231156 | ABCG2 | 0.664 | 0.534 | **0.016** | **0.009** | 0.393 | 0.641 | 0.811 | 0.888 | 0.266 | 0.812 | 0.827 |
| rs2231157 | ABCG2 | 0.427 | 0.425 | 0.875 | 0.961 | 0.891 | 0.902 | 0.537 | 0.930 | 0.443 | 0.136 | 0.262 |
| rs2231165 | ABCG2 | 0.259 | 0.154 | 0.666 | 0.509 | 0.909 | 0.753 | 0.545 | 0.782 | 0.785 | 0.074 | 0.341 |
| rs4148152 | ABCG2 | 0.065 | 0.051 | 0.092 | 0.097 | 0.262 | 0.738 | 0.133 | 0.337 | 0.0817 | 0.313 | 0.188 |
| rs112236246 | CES1 | 0.638 | 0.514 | 0.578 | 0.700 | 0.472 | 0.959 | 0.875 | 0.656 | 0.672 | 0.956 | 0.531 |
| **rs2244613#** | CES1 | 0.158 | 0.087 | 0.381 | 0.574 | 0.902 | 0.412 | 0.272 | 0.814 | 0.326 | 0.857 | 0.801 |
| rs2244614 | CES1 | 0.611 | 0.483 | 0.600 | 0.723 | 0.471 | 0.973 | 0.894 | 0.640 | 0.713 | 0.916 | 0.633 |
| rs2302719 | CES1 | 0.152 | 0.084 | 0.435 | 0.594 | 0.995 | 0.556 | 0.343 | 0.952 | 0.617 | 0.747 | 0.829 |
| rs3217164 | CES1 | 0.660 | 0.562 | 0.783 | 0.908 | 0.428 | 0.946 | 0.858 | 0.478 | 0.940 | 0.897 | 0.713 |
| rs3815583 | CES1 | 0.167 | **0.045** | 0.457 | 0.264 | 0.181 | 0.725 | 0.081 | 0.133 | 0.116 | 0.090 | 0.393 |
| rs56278207 | CES1 | 0.244 | 0.462 | 0.634 | 0.700 | **0.028** | **0.035** | 0.944 | 0.337 | 0.703 | 0.957 | 0.675 |
| rs12769205 | CYP2C19 | 0.140 | 0.107 | 0.781 | 0.761 | 0.986 | 0.733 | 0.155 | **0.007** | 0.319 | **0.031** | 0.286 |
| rs17885098 | CYP2C19 | 0.987 | 0.934 | 0.559 | 0.623 | 0.929 | 0.958 | 0.412 | 0.322 | 0.338 | 0.752 | 0.712 |
| rs3758580 | CYP2C19 | 0.140 | 0.107 | 0.781 | 0.761 | 0.986 | 0.733 | 0.155 | **0.007** | 0.319 | **0.031** | 0.286 |
| **rs4244285#** | CYP2C19 | 0.124 | 0.098 | 0.811 | 0.688 | 0.990 | 0.720 | 0.113 | **0.006** | 0.249 | **0.042** | 0.270 |
| **rs1065852#** | CYP2D6 | 0.940 | 0.978 | 0.315 | 0.447 | 0.984 | 0.963 | 0.765 | 0.229 | 0.566 | 0.439 | 0.329 |
| rs1080995 | CYP2D6 | 0.116 | 0.098 | 0.477 | 0.686 | 0.933 | 0.654 | 0.706 | 0.957 | 0.990 | 0.230 | 0.065 |
| rs1081003 | CYP2D6 | 0.800 | 0.897 | 0.424 | 0.547 | 0.883 | 0.894 | 0.596 | 0.349 | 0.442 | 0.349 | 0.400 |
| rs1135840 | CYP2D6 | 0.249 | 0.225 | 0.669 | 0.768 | 0.984 | 0.557 | 0.891 | 0.096 | 0.591 | 0.552 | 0.546 |
| rs16947 | CYP2D6 | 0.088 | 0.064 | 0.451 | 0.594 | 0.662 | 0.378 | 0.764 | 0.909 | 0.952 | 0.214 | 0.060 |
| **rs28371725#** | CYP2D6 | 0.536 | 0.383 | 0.592 | 0.300 | 0.947 | 0.833 | 0.929 | 0.931 | 0.862 | 0.836 | 0.241 |
| rs2291075 | SLCO1B1 | **0.002** | **0.002** | 0.138 | 0.354 | 0.735 | 0.856 | 0.127 | 0.154 | 0.079 | 0.528 | 0.212 |
| rs2291076 | SLCO1B1 | 0.739 | 0.926 | 0.588 | 0.608 | 0.828 | 0.940 | 0.880 | 0.406 | 0.294 | 0.726 | 0.945 |
| **rs2306283#** | SLCO1B1 | 0.416 | 0.667 | 0.990 | 0.920 | 0.866 | 0.768 | 0.352 | 0.367 | 0.549 | 0.601 | 0.632 |
| rs4149032 | SLCO1B1 | **0.017** | 0.108 | 0.421 | 0.552 | 0.250 | 0.804 | 0.312 | 0.942 | 0.793 | 0.495 | 0.559 |
| rs4149033 | SLCO1B1 | **0.031** | 0.060 | 0.602 | 0.732 | 0.095 | 0.707 | 0.382 | 0.254 | 0.624 | 0.281 | 0.517 |
| rs4149034 | SLCO1B1 | **0.011** | 0.079 | 0.411 | 0.583 | 0.346 | 0.958 | 0.271 | 0.850 | 0.881 | 0.505 | 0.424 |
| **rs4149056#** | SLCO1B1 | **0.017** | **0.044** | 0.990 | 0.801 | 0.984 | 0.537 | 0.154 | 0.107 | 0.533 | 0.430 | 0.080 |
| rs4149057 | SLCO1B1 | 0.670 | 0.984 | 0.551 | 0.542 | 0.848 | 0.990 | 0.685 | 0.415 | 0.348 | 0.720 | 0.886 |
| rs11045748 | SLCO1B7,SLCO1B1 | **0.035** | **0.020** | 0.156 | 0.112 | 0.132 | 0.390 | 0.102 | **0.047** | **0.023** | 0.073 | **0.034** |
| chr4:69879878 | UGT2A3,UGT2B7 | 0.630 | 0.722 | **0.039** | **0.021** | 0.333 | 0.436 | 0.874 | 0.4787 | 0.383 | 0.407 | 0.542 |
| rs115791839 | UGT2A3,UGT2B7 | 0.630 | 0.722 | **0.039** | **0.021** | 0.333 | 0.436 | 0.874 | 0.4787 | 0.383 | 0.407 | 0.542 |
| rs12233719 | UGT2B7 | 0.148 | 0.106 | 0.570 | 0.508 | 0.752 | 0.566 | 0.411 | 0.8879 | 0.106 | 0.570 | 0.910 |
| rs28365063 | UGT2B7 | 0.176 | 0.130 | 0.789 | 0.598 | 0.691 | 0.512 | 0.204 | 0.5882 | 0.060 | 0.575 | 0.960 |
| rs4257713 | UGT2B7 | 0.115 | 0.089 | 0.253 | 0.197 | 0.397 | 0.229 | 0.498 | 0.4739 | 0.501 | 0.567 | 0.472 |
| rs5013211 | UGT2B7 | 0.198 | 0.115 | 0.380 | 0.326 | 0.747 | 0.380 | 0.494 | 0.7056 | 0.200 | 0.959 | 0.931 |
| rs7438284 | UGT2B7 | 0.246 | 0.166 | 0.392 | 0.363 | 0.760 | 0.330 | 0.504 | 0.7335 | 0.190 | 0.878 | 0.967 |
| rs7658752 | UGT2B7 | 0.246 | 0.166 | 0.392 | 0.363 | 0.760 | 0.330 | 0.504 | 0.7335 | 0.190 | 0.878 | 0.967 |
| rs4646427 | CYP1A2 | 0.732 | 0.454 | 0.515 | 0.966 | **0.017** | **0.044** | 0.059 | 0.412 | **0.019** | 0.814 | 0.116 |
| rs1137115 | CYP2A6 | 0.839 | 0.683 | 0.699 | 0.522 | 0.445 | 0.353 | 0.950 | 0.641 | 0.600 | 0.218 | 0.486 |
| rs8192720 | CYP2A6 | 0.915 | 0.912 | 0.574 | 0.809 | 0.089 | 0.065 | 0.753 | 0.983 | 0.489 | 0.646 | 0.106 |
| rs8192725 | CYP2A6 | 0.762 | 0.604 | 0.740 | 0.499 | 0.409 | 0.326 | 0.828 | 0.961 | 0.506 | 0.195 | 0.434 |
| rs8192726 | CYP2A6 | 0.760 | 0.735 | 0.822 | 0.695 | 0.488 | 0.263 | 0.714 | 0.427 | 0.787 | 0.866 | 0.923 |
| rs2279342 | CYP2B6 | 0.069 | 0.075 | 0.210 | 0.116 | 0.377 | 0.653 | **0.007** | 0.124 | 0.343 | 0.968 | 0.615 |
| rs8192719 | CYP2B6 | 0.863 | 0.740 | 0.335 | 0.707 | 0.404 | 0.523 | 0.285 | 0.243 | 0.241 | 0.280 | 0.193 |
| rs34433978 | CYP2B6,CYP2A13 | 0.075 | 0.051 | 0.852 | 0.659 | 0.189 | 0.168 | **0.020** | **0.019** | 0.097 | **0.012** | **0.035** |
| rs35930845 | CYP2B6,CYP2A13 | 0.198 | 0.075 | 0.280 | 0.415 | 0.558 | 0.324 | 0.100 | 0.856 | 0.977 | 0.964 | 0.908 |
| rs3745275 | CYP2B6,CYP2A13 | 0.381 | 0.172 | 0.985 | 0.509 | 0.624 | 0.804 | 0.610 | 0.769 | 0.922 | 0.674 | 0.705 |
| rs3745276 | CYP2B6,CYP2A13 | 0.789 | 0.702 | 0.574 | 0.992 | 0.278 | 0.392 | 0.922 | 0.720 | 0.970 | 0.399 | 0.700 |
| rs3745277 | CYP2B6,CYP2A13 | 0.918 | 0.884 | 0.945 | 0.153 | 0.895 | 0.750 | 0.837 | 0.838 | 0.725 | 0.319 | 0.571 |
| rs434606 | CYP2B6,CYP2A13 | **0.028** | **0.014** | 0.105 | 0.137 | 0.136 | 0.370 | 0.195 | 0.869 | 0.700 | 0.386 | 0.675 |
| rs56156262 | CYP2B6,CYP2A13 | **0.016** | **0.014** | 0.976 | 0.799 | 0.342 | 0.259 | 0.147 | 0.256 | 0.242 | 0.105 | 0.113 |
| rs7249735 | CYP2B6,CYP2A13 | 0.739 | 0.425 | 0.696 | 0.699 | 0.224 | 0.336 | 0.787 | 0.477 | 0.624 | 0.589 | 0.890 |
| rs1058932 | CYP2C8 | 0.595 | 0.557 | 0.609 | 0.536 | 0.889 | 0.746 | 0.699 | 0.465 | 0.534 | 0.319 | 0.690 |
| rs11572078 | CYP2C8 | 0.590 | 0.553 | 0.825 | 0.388 | 0.868 | 0.708 | 0.748 | 0.420 | 0.544 | 0.252 | 0.632 |
| rs2071426 | CYP2C8 | 0.160 | 0.209 | 0.830 | 0.634 | 0.653 | 0.760 | 0.199 | 0.908 | 0.520 | 0.992 | 0.956 |
| rs2275622 | CYP2C8 | 0.295 | 0.255 | 0.688 | 0.331 | 0.654 | 0.553 | 0.209 | 0.234 | 0.151 | 0.117 | 0.324 |
| rs2229189 | CYP2J2 | 0.355 | 0.194 | 0.386 | 0.322 | 0.937 | 0.865 | 0.589 | 0.701 | 0.925 | 0.610 | 0.924 |
| rs2271800 | CYP2J2 | 0.884 | 0.732 | 0.852 | 0.757 | 0.569 | 0.411 | 0.196 | 0.361 | 0.199 | 0.352 | 0.138 |
| rs15524 | CYP3A5 | 0.489 | 0.503 | 0.534 | 0.270 | 0.189 | 0.256 | 0.645 | 0.964 | 0.051 | 0.120 | **0.037** |
| rs4646453 | CYP3A5,ZSCAN25 | 0.460 | 0.465 | 0.930 | 0.478 | 0.169 | 0.235 | 0.811 | 0.923 | 0.098 | 0.093 | **0.050** |
| rs2074900 | CYP4F2 | 0.467 | 0.853 | 0.309 | 0.538 | 0.054 | 0.066 | 0.734 | 0.843 | 0.827 | 0.744 | 0.277 |
| rs3093106 | CYP4F2 | 0.386 | 0.263 | 0.711 | 0.941 | 0.882 | 0.858 | 0.158 | 0.490 | 0.680 | 0.079 | 0.145 |
| rs3093160 | CYP4F2 | 0.386 | 0.263 | 0.711 | 0.941 | 0.882 | 0.858 | 0.158 | 0.490 | 0.680 | 0.079 | 0.145 |
| rs1867351 | SLC22A1 | 0.836 | 0.998 | 0.826 | 0.901 | 0.979 | 0.949 | 0.407 | 0.638 | 0.370 | 0.853 | 0.789 |
| rs2282143 | SLC22A1 | 0.732 | 0.489 | 0.439 | 0.873 | 0.349 | 0.396 | 0.420 | 0.571 | 0.492 | 0.882 | 0.732 |
| rs35854239 | SLC22A1 | 0.566 | 0.514 | 0.749 | 0.684 | 0.948 | 0.851 | 0.993 | 0.199 | 0.079 | 0.999 | 0.513 |
| rs4646273 | SLC22A1 | 0.815 | 0.983 | 0.921 | 0.779 | 0.973 | 0.878 | 0.220 | 0.554 | 0.532 | 0.706 | 0.997 |
| rs622591 | SLC22A1 | 0.658 | 0.947 | 0.507 | 0.143 | 0.527 | 0.346 | 0.117 | **0.027** | 0.623 | 0.191 | 0.449 |
| rs628031 | SLC22A1 | 0.424 | 0.413 | 0.726 | 0.528 | 0.958 | 0.947 | 0.944 | 0.124 | 0.118 | 0.913 | 0.713 |
| rs683369 | SLC22A1 | 0.597 | 0.625 | 0.431 | 0.324 | 0.463 | 0.358 | 0.564 | 0.807 | 0.608 | 0.520 | 0.706 |
| rs4148323 | UGT1A1 | **0.008** | **0.017** | 0.078 | 0.152 | 0.974 | 0.919 | 0.631 | 0.757 | 0.748 | 0.630 | 0.760 |
| rs2302538 | UGT1A1,3-10 | 0.071 | 0.245 | 0.630 | 0.827 | 0.461 | 0.458 | 0.376 | 0.857 | 0.849 | 0.637 | 0.874 |
| rs4148327 | UGT1A1,3-10 | **0.013** | **0.030** | 0.245 | 0.161 | 0.570 | 0.659 | 0.185 | 0.654 | 0.257 | 0.244 | 0.300 |
| rs12466997 | UGT1A3-10 | 0.947 | 0.895 | 0.595 | 0.147 | 0.618 | 0.751 | 0.522 | 0.796 | 0.442 | **0.046** | 0.346 |
| rs2361501 | UGT1A3-10 | 0.853 | 0.752 | 0.812 | 0.534 | 0.486 | 0.384 | 0.701 | 0.576 | 0.953 | 0.519 | 0.675 |
| rs869283 | UGT1A5-10 | 0.702 | 0.823 | 0.578 | 0.224 | 0.990 | 0.766 | 0.604 | 0.387 | 0.863 | 0.467 | 0.802 |
| rs7563561 | UGT1A7-10 | 0.121 | 0.173 | **0.035** | **0.033** | 0.464 | 0.439 | 0.333 | 0.940 | 0.850 | 0.917 | 0.837 |
| rs7608175 | UGT1A7-10 | 0.121 | 0.173 | **0.035** | **0.033** | 0.464 | 0.439 | 0.333 | 0.940 | 0.850 | 0.917 | 0.837 |
| rs7586110 | UGT1A8-10 | **0.007** | **0.017** | **0.025** | **0.028** | 0.531 | 0.484 | 0.170 | 0.932 | 0.793 | 0.633 | 0.870 |

ABCB1: ATP-binding cassette subfamily B member 1; CES1: Carboxylesterase 1; ABCC2: ATP Binding Cassette Subfamily C Member 2; ABCG2: ATP Binding Cassette Subfamily G Member 2; CYP2C19: Cytochrome P450 Family 2 Subfamily C Member 19; CYP2D6: Cytochrome P450 Family 2 Subfamily D Member 6; SLCO1B1: Solute Carrier Organic Anion Transporter Family Member 1B1; SLCO1B7: Solute Carrier Organic Anion Transporter Family Member 1B7; UGT2B7: UDP Glucuronosyltransferase Family 2 Member B7; UGT2A3: UDP Glucuronosyltransferase Family 2 Member A3; CYP1A2: Cytochrome P450 Family 1 Subfamily A Member 2; CYP2A6: Cytochrome P450 Family 2 Subfamily A Member 6; CYP2B6: Cytochrome P450 Family 2 Subfamily B Member 6; CYP2A13: Cytochrome P450 Family 2 Subfamily A Member 13; CYP2C8: Cytochrome P450 Family 2 Subfamily C Member 8; CYP2J2: Cytochrome P450 Family 2 Subfamily J Member 2;CYP3A5: Cytochrome P450 Family 3 Subfamily A Member 5; ZSCAN25: Zinc Finger And SCAN Domain Containing 25; CYP4F2: Cytochrome P450 Family 4 Subfamily F Member 2; SLC22A1: Solute Carrier Family 22 Member 1; UGT1A1: UDP Glucuronosyltransferase Family 1 Member A1; UGT1A3: UDP Glucuronosyltransferase Family 1 Member A3; UGT1A4: UDP Glucuronosyltransferase Family 1 Member A4; UGT1A5: UDP Glucuronosyltransferase Family 1 Member A5; UGT1A6: UDP Glucuronosyltransferase Family 1 Member A6; UGT1A7: UDP Glucuronosyltransferase Family 1 Member A7; UGT1A8: UDP Glucuronosyltransferase Family 1 Member A8; UGT1A9: UDP Glucuronosyltransferase Family 1 Member A9; UGT1A10: UDP Glucuronosyltransferase Family 1 Member A10; C_max_: maximum plasma concentration; T_max_: time to peak concentration; t_½_: half-life; IIa2h: value of anti-IIa at 2 h after dosing; APTT2h: value of activated partial thromboplastin time at 2 h after dosing; ∆ APTT2h: fold change in APTT at post-dose 2h to pre-dose 0h; PT2h: value of prothrombin time at 2 h after dosing; ∆ PT2h: fold change in PT at post-dose 2h to pre-dose 0h; A1: minor allele; A2: non- minor allele;

#The SNP was detected in reported pharmacogenomic studies of dabigatran
